# *Legionella pneumophila* Secretes a Mitochondrial Carrier Protein during Infection

**DOI:** 10.1371/journal.ppat.1002459

**Published:** 2012-01-05

**Authors:** Pavel Dolezal, Margareta Aili, Janette Tong, Jhih-Hang Jiang, Carlo M. Marobbio, Sau fung Lee, Ralf Schuelein, Simon Belluzzo, Eva Binova, Aurelie Mousnier, Gad Frankel, Giulia Giannuzzi, Ferdinando Palmieri, Kipros Gabriel, Thomas Naderer, Elizabeth L. Hartland, Trevor Lithgow

**Affiliations:** 1 Department of Biochemistry and Molecular Biology, Monash University, Clayton, Australia; 2 Department of Parasitology, Charles University, Prague, Czech Republic; 3 Department of Microbiology and Immunology, University of Melbourne, Parkville, Australia; 4 Department of Pharmaco-Biology, Laboratory of Biochemistry and Molecular Biology, University of Bari, Bari, Italy; 5 Department of Tropical Medicine, 1st Faculty of Medicine, Charles University in Prague and Faculty Hospital Bulovka, Prague, Czech Republic; 6 Centre for Molecular Microbiology and Infection, Division of Cell and Molecular Biology, Imperial College London, London, United Kingdom; Yale University School of Medicine, United States of America

## Abstract

The Mitochondrial Carrier Family (MCF) is a signature group of integral membrane proteins that transport metabolites across the mitochondrial inner membrane in eukaryotes. MCF proteins are characterized by six transmembrane segments that assemble to form a highly-selective channel for metabolite transport. We discovered a novel MCF member, termed *Legionella* nucleotide carrier Protein (LncP), encoded in the genome of *Legionella pneumophila*, the causative agent of Legionnaire's disease. LncP was secreted via the bacterial Dot/Icm type IV secretion system into macrophages and assembled in the mitochondrial inner membrane. In a yeast cellular system, LncP induced a dominant-negative phenotype that was rescued by deleting an endogenous ATP carrier. Substrate transport studies on purified LncP reconstituted in liposomes revealed that it catalyzes unidirectional transport and exchange of ATP transport across membranes, thereby supporting a role for LncP as an ATP transporter. A hidden Markov model revealed further MCF proteins in the intracellular pathogens, *Legionella longbeachae* and *Neorickettsia sennetsu*, thereby challenging the notion that MCF proteins exist exclusively in eukaryotic organisms.

## Author Summary

Mitochondrial carrier proteins evolved during endosymbiosis to transport substrates across the mitochondrial inner membrane. As such the proteins are associated exclusively with eukaryotic organisms. Despite this, we identified putative mitochondrial carrier proteins in the genomes of different intracellular bacterial pathogens, including *Legionella pneumophila*, the causative agent of Legionnaire's disease. We named the mitochondrial carrier protein from *L. pneumophila* LncP and determined that the protein is translocated into host cells during infection by the bacterial Dot/Icm type IV secretion system. From there, LncP accesses the classical mitochondrial import pathway and is incorporated into the mitochondrial inner membrane as an integral membrane protein. Remarkably, LncP crosses five biological membranes to reach its final location. Biochemically, LncP is a unidirectional nucleotide transporter similar to Aac1 in yeast. Although not essential for intracellular replication, the high carriage rate of *lncP* among isolates of *L. pneumophila* suggests that the ability of the pathogen to manipulate mitochondrial ATP transport assists survival of the bacteria in an intracellular environment.

## Introduction

*Legionella pneumophila* is an intracellular pathogen and the major causative agent of Legionnaire's disease, an acute form of pneumonia. The ability of the bacteria to replicate in environmental protozoa such as amoebae has equipped the bacteria with the capacity to replicate in human alveolar macrophages, leading to lung inflammation and disease [1], [2]. Within macrophages and amoebae, the bacteria replicate within a membrane bound vacuole, block phagolysosome fusion and intercept vesicles trafficking in the secretory pathway [3], [4]. Mitochondria are also transiently recruited to the *L. pneumophila* intracellular compartment [5]. The membrane of the mature *Legionella*-containing vacuole (LCV) shares many characteristics with membrane of the rough endoplasmic reticulum, reviewed in [6], [7] but interactions with the endocytic pathway are also evident [8]. Therefore formation of the intracellular replicative niche of *L. pneumophila* results from extensive remodelling of the intracellular vacuole and multiple interactions with vesicle trafficking pathways within the host cell [8], [9].

The formation of the LCV relies on a functional bacterial Dot/Icm Type IVB secretion system, which delivers at least 275 effectors into the host cell cytosol [10]–[13]. The effectors target multiple host cell functions including GTPase activity, ubiquitination, phosphoinositide metabolism, eukaryotic protein translation, autophagy and apoptosis, reviewed in [6], [14]–[17]. Many groups of effectors have overlapping and somewhat redundant activities making the use of reverse bacterial genetics to identify gene function difficult. Instead, many investigators have applied cell biology and protein biochemistry techniques to understand the biochemical activity of Dot/Icm effectors and their possible role during LCV formation and *L. pneumophila* intracellular replication [18]–[21].

Genomics has revealed that a substantial number of Dot/Icm effectors share similarity with eukaryotic proteins [22]. For example, a large group of effectors contain multiple ankyrin repeat domains [23] and another group share similarity with F-box and U-box proteins involved in protein ubiquitination [24]–[26]. One effector termed LegS2 shares amino acid sequence similarity with eukaryotic sphingosine-1-phosphate lyases and is targeted to mitochondria during infection [27], although the importance of this targeting to LegS2 function is unknown.

In this study, we discovered that the genome of *L. pneumophila* strain 130b encodes a putative member of the Mitochondrial Carrier Family (MCF), termed LncP for *Legionella* nucleotide carrier Protein. MCF proteins are a signature family of eukaryotic proteins that evolved in the course of endosymbiosis, ultimately giving rise to mitochondria [28]. MCF proteins are found in the broadest distribution of eukaryotes, including humans, yeast, plants and parasites such as trypanosomes and amoebae [29]–[32]. In humans, yeast and other eukaryotes, MCF proteins are synthesized in the cytoplasm and enter mitochondria via a defined “carrier pathway”. The proteins are chaperoned through the cytosol by Hsp70/Hsp90 and delivered to the Tom70 receptor on the mitochondrial surface [33]. After threading through the channel in the outer mitochondrial membrane, unfolded MCFs are bound by the TIM9:10 chaperone in the intermembrane space and then assembled into the mitochondrial inner membrane by the TIM22 complex (reviewed in [34]–[37]). Here we found that LncP was translocated into host cells by the Dot/Icm type IV secretion system and transported into the mitochondrial inner membrane by the mitochondrial TIM9:10 chaperones and the TIM22 complex. A yeast model system and biochemical transport assays suggested that LncP mediated the unidirectional transport of ATP. In this otherwise exclusively eukaryotic group of proteins, LncP is the first example of a MCF member from bacteria that may contribute to the persistence of *L. pneumophila* within eukaryotic cells.

## Results

### *Legionella pneumophila* Encodes a Putative Mitochondrial Carrier Protein

**Figure 1:**
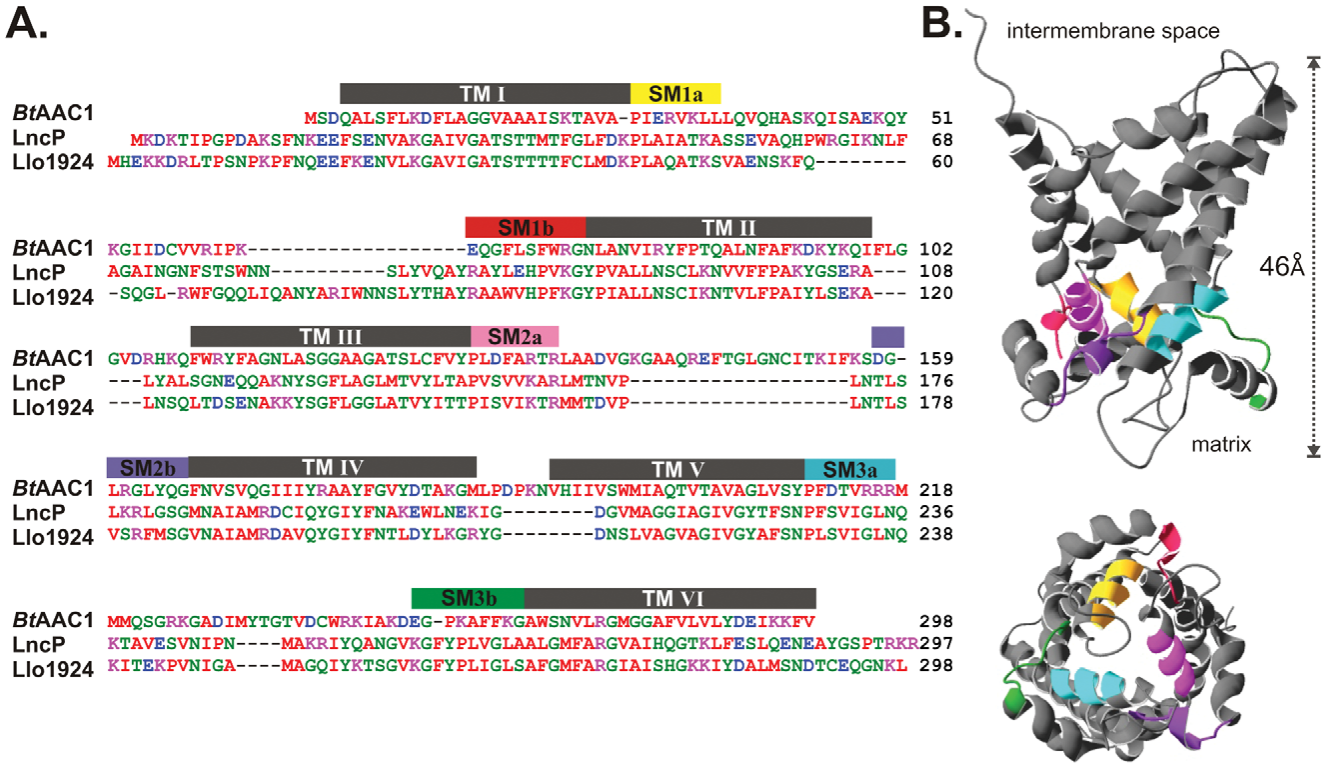
A mitochondrial carrier protein in *Legionella*. (A) Sequence alignment of LncP from *L. pneumophila* and Llo1924 from *L. longbeachae* with the ADP/ATP carrier from *Bos taurus*. Amino acid residues are colored red (hydrophobic), blue (acidic), magenta (basic), green (polar) and the six predicted transmembrane segments shown. Conservation is seen through the predicted transmembrane segments and in the three-fold repeated signature motif (labeled SM1a-SM1b, SM2a-SM2b, SM3a-SM3b), all of which are characteristic of all members of the mitochondrial carrier protein family [40], [41]. (B) The three-dimensional structure of the ADP/ATP carrier from *B. taurus* (PDB: 1OKC), with the three-fold repeated signature motif color-coded as shown in Figure 1A. The folded protein has a “height” of 46 Å and the maximum “width” dimension is 41 Å.

When the UniProt data set of protein sequences was screened with a hidden Markov model for mitochondrial carrier family (MCF) proteins, an expected number of MCF proteins were detected in mammals, plants and fungi [38]–[42] and a smaller number in protists such as *Entamoeba histolytica* [32]. Unexpectedly, a handful of protein sequences was also retrieved from bacteria. Two of these were encoded in the genome of the intracellular pathogen *Neorickettsia sennetsu*, the causative agent of Sennetsu fever [43], [44]. Three other carriers were encoded in the genome of *L. longbeachae* (Llo1924, Llo3082 and Llo1358), with Llo1924 having a homolog (sequence identity of 57%; Figure 1A), encoded in the genome of the related pathogen *L. pneumophila* strain 130b (open-reading frame LPW_31961) [45], [46]. The putative MCF protein from *L. pneumophila* was subsequently termed LncP.

The crystal structure of the prototypical MCF, the adenine nucleotide transporter from mammals, shows that the protein has six transmembrane segments that are embedded in the mitochondrial inner membrane [47]. Bioinformatic analysis indicated that the amino acid sequences of Llo1924 and LncP had six predicted transmembrane segments and a three-fold repeated signature motif (Figure 1A) which are the essential characteristics of members of the MCF (Figure 1B) [38]–[42]. MCF proteins differ to nucleotide carriers in the inner membranes of the Chlamydiales and the Rickettsiales, which represent different family of proteins, referred to as TLC ATP/ADP transporters (PF03219) [48], [49]. This latter group is of bacterial origin, and has spread from chlamydial ancestors to other classes of bacteria and to chloroplasts via lateral gene transfer events. TLC ATP/ADP transporters contain twelve transmembrane segments and their nucleotide exchange properties do not require membrane potential [50].

### LncP Is Targeted to Mitochondria during Infection in a Dot/Icm T4SS-dependent Manner

**Figure 2:**
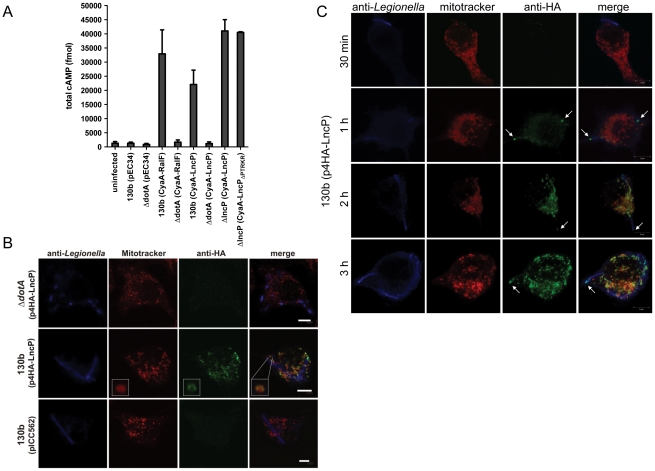
LncP is translocated into macrophages by the Dot/Icm T4SS. (A) THP-1 macrophages were left uninfected or infected with derivatives of *L. pneumophila* 130b carrying the pEC34 vector or expressing the indicated Cya hybrid proteins. Following infection for 1 hour, macrophages were lysed and total intracellular cAMP was measure by ELISA. Results are expressed as fmol cAMP and are the mean ± standard deviation of three independent experiments, each performed in duplicate. Note Cya-LncP_ΔPTRKR_ is a truncated protein lacking the C-terminal residues (PTRKR) of LncP. (B) Immortalized macrophages from C57BL/6 mice were infected with derivatives of *L. pneumophila* 130b for 5 h as indicated. Bacteria were visualized using anti-*Legionella* antibodies (blue) 4HA-LncP was visualized with antibodies to HA (green). Prior to fixation, cells were stained with MitoTracker Red. Cells were viewed by confocal microscopy under a 100× objective. White scale bars represent 5 µm. (C) Immortalized macrophages from C57BL/6 mice were infected with derivatives of *L. pneumophila* 130b for 30 min, 1 h, 2 h or 3 h as indicated, stained as above, and viewed by confocal microscopy under a 100× objective. White scale bars represent 5 µm. Arrows indicate LncP at the poles of the bacterial cell.

Many eukaryotic-type proteins from *L. pneumophila* are translocated into infected cells via the Dot/Icm type IV secretion system. To determine if LncP was a Dot/Icm effector, we generated a translational fusion of the calmodulin-dependent adenylate cyclase from *Bordetella pertussis* (CyaA), with the N-terminus of LncP (Cya-LncP). The Cya-LncP fusion construct was introduced into wild type *L. pneumophila* 130b or a *dot/icm* (*dotA*) mutant [51]. Upon infection of THP-1 macrophages, Cya-LncP translocation was detected by increased cyclic AMP (cAMP) production at levels similar to the positive control (Cya-RalF) (Figure 2A). This translocation was dependent on *dotA* indicating that LncP is a Dot/Icm effector. Compared to eukaryotic MCF members, LncP carries a short amino acid extension at the C-terminus (Figure 1A). As the secretion signal for many Dot/Icm effectors lies in the C-terminus of the protein [52], [53], we tested whether this region contained a Dot/Icm secretion signal, however deletion of the C-terminal amino acid residues PTRKR had no effect on Dot/Icm dependent translocation (Figure 2A).

To determine if LncP localized to mitochondria during infection of macrophages, we generated a 4HA epitope-tagged version of LncP for expression in *L. pneumophila*. The resulting expression plasmid, p4HA-LncP, was transformed into wild type *L. pneumophila* 130b and the *dotA* mutant. Upon infection of macrophages for 5 h with 130b carrying p4HA-LncP, anti-HA staining co-localized extensively with Mitotracker red in infected cells (Figure 2B). Anti-HA staining was not observed in macrophages infected with *L. pneumophila* 130b carrying the empty vector, pICC562, or in macrophages infected with the *dotA* mutant carrying p4HA-LncP (Figure 2B). Similar results were observed upon *L. pneumophila* infection of HeLa cells (Figure S1). We detected increasing amounts of LncP associated with mitochondria over time (Figure 2C) and at earlier time points, we frequently observed LncP staining at the poles of the bacterial cell where the Dot/Icm secretion system is believed to be located (Figure 2C). Altogether, this demonstrated that LncP was localized to mitochondria during *L. pneumophila* infection and this event relied upon a functional *dot/icm* system.

**Figure 3:**
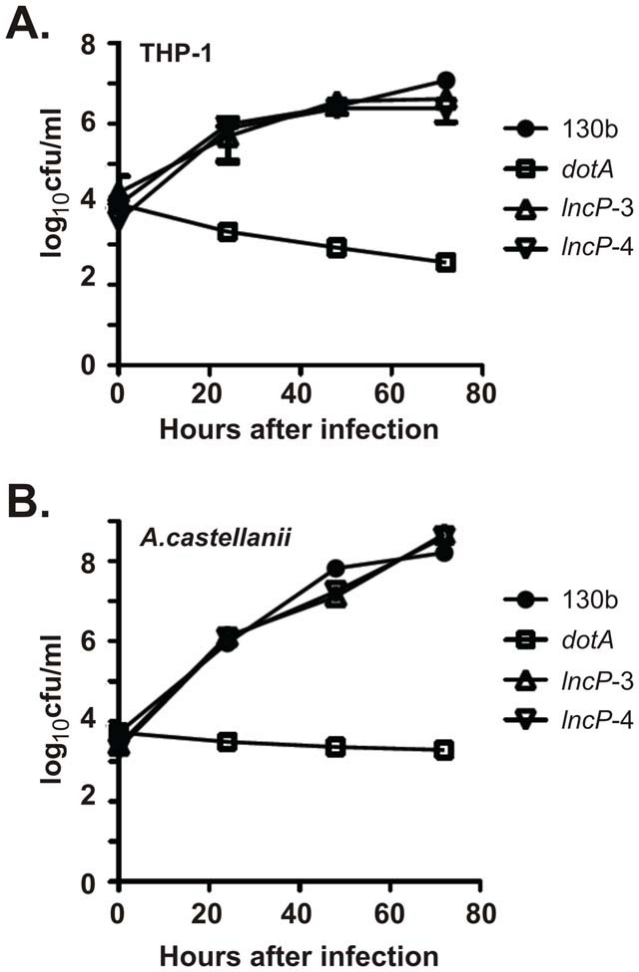
Mutant *L. pneumophila* lacking LncP replicate proficiently in host cells. Two independent mutants of *L. pneumophila* 130b lacking LncP (*lncP-*3 and *lncP-*4) were tested, along with a *dotA* mutant lacking the Dot/Icm T4SS. Replication of *L. pneumophila* 130b (•),*lncP-*3 (Δ),*lncP-*4 (▿) and *dotA* (□) within the macrophage cell-line THP-1 (A) and *A. castellanii* (B) is shown. Results are expressed as the log_10_CFU of viable bacteria present in the extracellular medium (and associated with cells for THP-1) at specific time points after inoculation, mean ± standard deviation of at least three independent experiments from duplicate wells.

Many genes encoding Dot/Icm effectors are dispensable for intracellular replication due to functional redundancy [9], reviewed in [6]. Likewise here, the gene encoding LncP was not required for *L. pneumophila* 130b intracellular replication in THP-1 macrophages (Figure 3A) or in the model amoeba, *Acanthamoeba castellanii* (Figure 3B). However, PCR screening of 37 distinct *L. pneumophila* isolates detected the gene encoding LncP in 28 of these strains (Table S1). The high carriage rate (∼75%) of the *lncP* gene among *L. pneumophila* strains strongly suggests LncP provides a competitive advantage during interactions with host cells.

### LncP Is an Integral Mitochondrial Inner Membrane Protein

**Figure 4:**
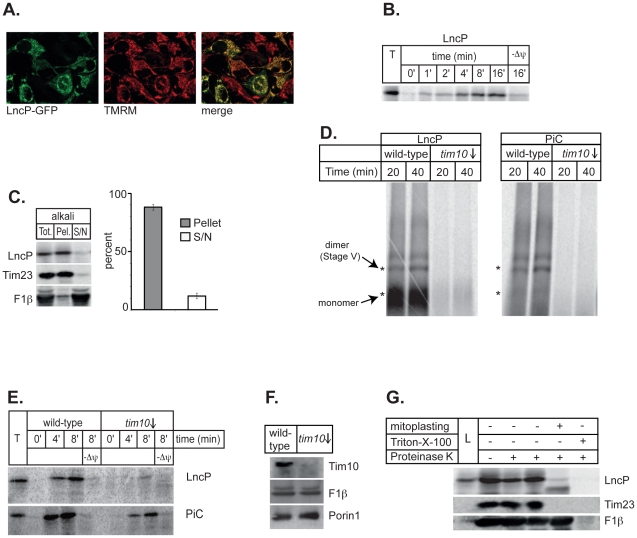
LncP is transported to the mitochondrial inner membrane. (A) HeLa cells were transformed to express LncP-GFP or a control plasmid. The LncP-GFP cells were co-stained with tetramethylrhodamine methyl ester (TMRM) and viewed by confocal microscopy. The merge shows the mitochondrial localization of LncP-GFP (B) Mitochondria (50 µg protein) from wild-type yeast cells were incubated with [^35^S]-labeled LncP. After the indicated time at 25°C, mitochondria were isolated, treated with Proteinase K to degrade protein that had not been imported, and analyzed by SDS-PAGE and fluorography. “T” represents non-Proteinase K treated control. “-Δψ_m_” indicates a sample where the mitochondria were pre-incubated with inhibitors and uncouplers to deplete the transmembrane potential (see Methods) (C) Mitochondria (100 µg protein) from wild-type cells were incubated with [^35^S]-labeled LncP. After 20 minutes at 25°C, mitochondria were isolated, extracted with 0.1 M Na2CO3 and the membrane-containing pellet (“Pel”) and extracted proteins in the supernatant (“S/N”) analyzed by SDS-PAGE and fluorography and immunoblot against a known membrane embedded protein (Tim23) and a non-membrane embedded protein matrix localized protein (F_1_β). A sample of mitochondria prior to extraction and representing the total amount (“Tot.”) is shown for comparison. The right-hand panel shows the percentage distribution of LncP in the pellet and supernatant fractions after 5 repeat experiments ± standard error. (D) Mitochondria (50 µg protein) from wild-type and Tim10 depleted (*tim10*↓) yeast cells were resuspended in isotonic import buffer and incubated with [^35^S]-labeled LncP and PiC. After the indicated time at 25°C, mitochondria were isolated, solubilized in digitonin and analyzed by BN-PAGE and fluorography. Asterisk indicates bands formed by folded carrier proteins. The lower asterisk represents the folded monomer and the upper asterisk represents assembled carrier dimers (Stage V) (E) Mitochondria (50 µg protein) from wild-type yeast or from *tim10*↓ yeast depleted of Tim10 were incubated with [^35^S]-labeled LncP or PiC. After the indicated time at 25°C, the mitochondria were treated with Proteinase K and then analysed by SDS-PAGE and fluorography. “-Δψ_m_” indicates a sample where the mitochondria were pre-incubated with inhibitors and uncouplers to deplete the transmembrane potential (see Methods). (F) Control western blots with mitochondria isolated from wild-type and *tim10*↓ cells respectively showing that Tim10 has been selectively depleted. (G) The localization of LncP within mitochondria after import was determined using a sequential proteolysis assay. After import of [^35^S]-labeled LncP at 25°C for 20 minutes, mitochondria were treated with hypotonic buffer to induce mitoplasting, or Triton-X-100 to rupture both membranes and Proteinase K (50 µg/mL) as indicated (see Methods). “L” is lysate only without mitochondria to show size of unimported protein. The control proteins, the inner membrane embedded protein (Tim23) and a non-membrane embedded protein matrix localized protein (F_1_β) were detected by immunoblot on the same membrane.

Fluorescence microscopy confirmed that GFP-LncP was targeted to mitochondria when expressed ectopically in HeLa cells (Figure 4A). This substantiates a model whereby cytosolic LncP can access the mitochondrial import machinery in mammalian cells. To test whether LncP was imported by mitochondria, the putative MCF protein was translated *in vitro* and incubated with mitochondria isolated from yeast. This represents the best experimental system to characterize the pathway by which LncP is imported into mitochondria. LncP was imported into mitochondria and protected from Proteinase K treatment showing that it is not imported into the mitochondrial outer membrane (Figure 4B). Import of mitochondrial carrier proteins is reliant on a membrane potential across the inner membrane. Here pretreatment of mitochondria with CCCP, that dissipates the transmembrane potential (Δψ_m_), also inhibited LncP import (Figure 4B, “-Δψ”). Imported LncP behaved as an integral inner membrane protein similar to Tim23, being largely resistant to alkali extraction, unlike the non-membrane embedded, matrix targeted protein, F_1_β (Figure 4C).

The TIM9:10 chaperone characteristically binds carrier proteins during the initial phase of their assembly in the inner mitochondrial membrane. Blue-native (BN)-PAGE analysis of imported phosphate carrier PiC (Figure 4D) and Aac1 (data not shown) showed intermediate forms of the carrier during its import pathway and final assembly as a mature dimer complex. Folded PiC mostly existed as the dimeric (Stage V) form with only a small amount of folded monomer detected. LncP was also assembled in mitochondria efficiently but much of the folded protein accumulated as monomeric protein, possibly because there was no pre-existing LncP in mitochondria with which imported LncP could oligomerise. The folding of carrier proteins is dependent on the TIM9:10 chaperone [54]–[56]. Mitochondria from a *tim10* “shut-down” strain were not able to assemble LncP or PiC into complexes detectable by BN-PAGE (Figure 4D). Consistent with this finding, mitochondria from a *tim10* “shut-down” strain, imported both PiC and LncP to a protease protected location at a greatly reduced efficiency (Figure 4E). When ImageQuant was used to compare the band intensities in lanes from wild-type and *tim10* mutant mitochondria, the percentage decrease of import for both PiC and LncP was between 20% and 35% of wild-type (data not shown). In order to show the localization of mitochondrial proteins unambiguously it is possible to sequentially rupture the mitochondrial outer membrane (mitoplasting) or both membranes and test for sensitivity to protease digestion. Since these protease treatments are sensitive to rough handling, the digestion was performed in duplicate. LncP was degraded by Proteinase K after rupture of the outer membrane (Figure 4G). This characteristic is consistent with that of Tim23, an integral inner membrane protein with domains exposed to the intermembrane space. The matrix targeted protein, F_1_β was not degraded by Proteinase K unless the inner membrane was also ruptured by the addition of detergent (Figure 4G). Slight changes in band intensity from lane to lane were not significant upon repetition, rather protease treatment drastically altered the levels of susceptible proteins such as after mitoplasting or treatment with detergent (Figure 4G).

### LncP Is a Nucleotide Carrier Protein

**Figure 5:**
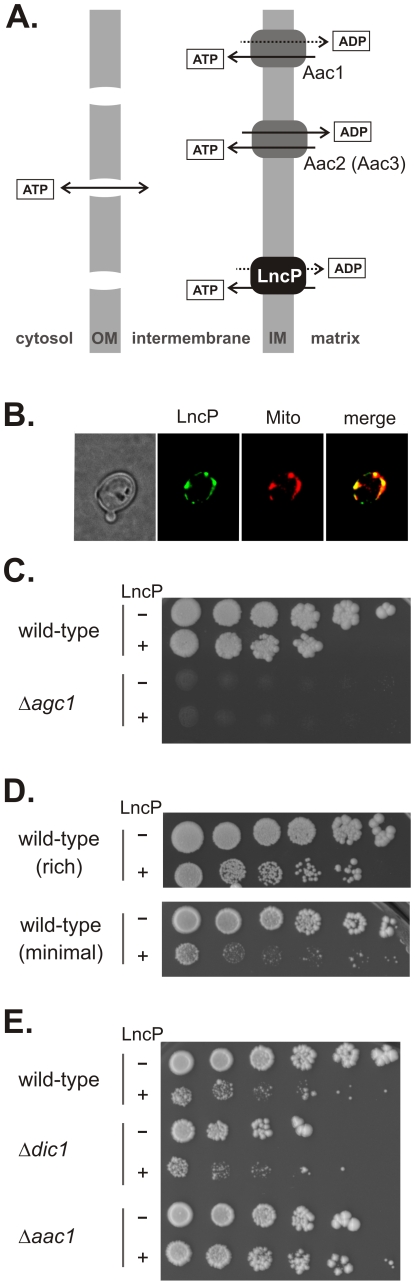
LncP generates a dominant negative phenotype, dependent on Aac1 ATP transport activity. (A) Yeast cells express three dominant carriers for adenine nucleotide transport: Aac1, Aac2 and Sal1 in the mitochondrial inner membrane (IM). Aac3 is an isoform expressed under anaerobic conditions [94]. The outer membrane (OM) of mitochondria is permeable to ATP due to the pores formed by VDAC. (B) Yeast cells transformed to express LncP-GFP were co-stained with MitoTracker Red and visualized by fluorescence microscopy. The merge shows the mitochondrial localization of LncP-GFP. (C) Yeast mutants, each lacking one member of the carrier protein family were transformed with either a plasmid encoding LncP (+) or the control (-) plasmid. The transformed cells were plated on selective medium and scored for growth using five-fold serial dilutions. As an example, the Δ*agc1* mutant is shown: Agc1 is an amino acid transporter which acts both as a glutamate uniporter and as an aspartate-glutamate exchanger; while viable on plates containing glycerol as a carbon source, the Δ*agc1* mutant cells form only microcolonies before arresting growth. Expression of LncP does not support glutamate-aspartate transport and so does not rescue this phenotype. (D) Wild-type cells transformed with either a plasmid encoding LncP (+) or the control (−) plasmid were plated on YPD medium with glucose as a carbon source (rich) or SD semi-synthetic medium with glucose as a carbon source (minimal) and scored for growth using five-fold serial dilutions. The number of colonies represents cell viability. (E) Yeast mutants, each lacking a distinct carrier protein, were transformed with either the plasmid encoding LncP (+) or the control (−) plasmid and scored for growth using five-fold serial dilutions. The Δ*dic1* mutant lacks the dicarboxylic acid transporter and is representative of carrier mutants in showing the same dominant-negative phenotype as wild-type cells. Only in the Δ*aac1* mutants is this phenotype suppressed.

*Saccharomyces cerevisiae* encodes 35 mitochondrial carrier proteins, including four proteins that can transport ATP: Aac1, Aac2, Aac3 and Sal1 [57] (Figure 5A). Yeast is a powerful model system to study cellular phenotypes, and fluorescence microscopy showed that ectopically expressed LncP is targeted to mitochondria in yeast (Figure 5B). Mutant yeast strains, each lacking one of these 35 carriers were transformed with a plasmid-based LncP expression construct and the transformed cells tested for growth complementation. The mutants were scored under conditions where characteristic growth defects were known. However no complementation was observed upon LncP expression in any of the mutants tested. For example, Δ*agc1* mutant cells lacking the amino acid transporter Agc1 form only microcolonies on rich medium with glycerol as a carbon source; expression of LncP did not complement this growth defect (Figure 5C). However, we noted a dominant-negative phenotype from expression of LncP in wild-type cells which represented a 5-fold loss in viability on rich growth medium, exacerbated to ∼500-fold loss of viability on minimal medium (Figure 5D). We therefore screened the carrier mutant collection for mutants resistant to this LncP-induced inhibition of cell viability. Only the Δ*aac1* mutant was resistant to the dominant-negative effect of LncP expression (Figure 5E). In yeast, Sal1 is a Ca^2+^-dependent ATP-import carrier that co-transports ATP and Mg^2+^ into the matrix during growth on glucose [58], [59], and the Aac1 transporter balances this effect by ATP export. The most likely explanation for the Aac1-dependent dominant-negative effect of LncP expression is that combined export of ATP from the matrix by LncP and Aac1 leads to a growth defect. Thus, yeast can tolerate the expression of Aac1 or LncP, but not both of these carrier proteins.

**Figure 6:**
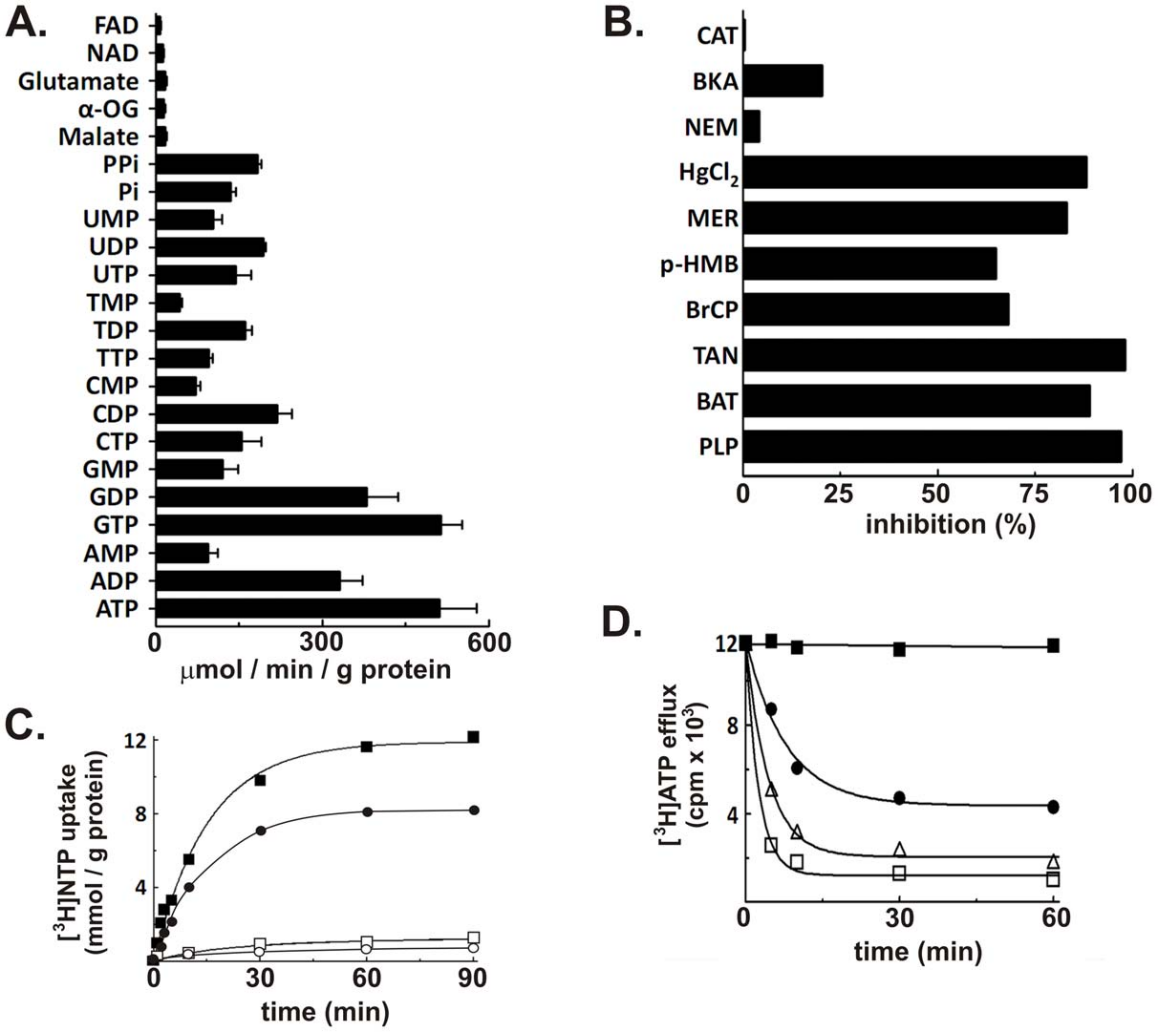
LncP is a nucleotide carrier with unique properties. (A) Liposomes reconstituted with LncP were preloaded internally with various substrates (concentration, 10 mM). Transport was started by the addition of 0.2 mM [^3^H]ATP and terminated after 2 min. Values are means ± S.D. of at least three independent experiments. α-OG, α-oxoglutarate; Pi, phosphate; PPi, pyrophosphate. (B) Proteoliposomes were preloaded internally with 10 mM ATP and transport was initiated by adding 0.2 mM [^3^H]ATP. The reaction time was 2 min. Thiol reagents were added 2 min before the labeled substrate; the other inhibitors were added together with the labeled substrate. The final concentrations of the inhibitors were 20 mM (PLP, pyridoxal-5′-phosphate; BAT, bathophenanthroline), 0.2 mM (*p*-HMB, *p*-hydroxymercuribenzoate; MER, mersalyl), 1 mM (NEM, *N*-ethylmaleimide), 0.2% (TAN, tannic acid), 0.2 mM (BrCP, bromcresol purple), 25 µM (HgCl_2_, mercuric chloride) and 10 µM (BKA, bongkrekic acid; CAT, carboxyatractyloside). The extent of inhibition (%) from representative experiments is given. (C) Uptake of [^3^H]ATP (▪, □) and [^3^H]GTP (•, ○) into liposomes reconstituted with LncP. 1 mM [^3^H]ATP or [^3^H]GTP was added to proteoliposomes containing 10 mM ATP or GTP, respectively (exchange, filled shapes), or 10 mM NaCl and no substrate (uniport, open shapes). Similar results were obtained in three independent experiments. (D) Efflux of [^3^H]ATP from LncP proteoliposomes. The internal substrate (2 mM ATP) was labeled by carrier-mediated exchange equilibration. After removal of the external substrate by Sephadex G-75, the efflux of [^3^H]ATP was started by adding buffer A alone (filled circles), 5 mM ATP, 20 mM pyridoxal-5′-phosphate and 10 mM bathophenanthroline in buffer A (filled squares), 5 mM ATP in buffer A (open squares) or 5 mM phosphate (open triangles). Similar results were obtained in three independent experiments.

In order to measure directly substrate transport catalyzed by LncP, purified recombinant protein was reconstituted into liposomes. LncP transported nucleotides, phosphate and pyrophosphate, with a strong preference for ATP and GTP (Figure 6A). The kinetic constants of purified reconstituted LncP were determined by measuring the initial transport rate at various external [^3^H]ATP or [^3^H]GTP concentrations in the presence of a fixed saturating internal concentration of ATP or GTP, respectively. The transport affinities (K_m_) of LncP for ATP and GTP were 190±37 and 183±32 µM, respectively. The average V_max_ values for ATP and GTP were 926±216 and 688±213 µmol/min x g of protein, respectively (mean values of 4 experiments).

Powerful inhibitors of the well-characterized ADP/ATP carrier, which transports only ADP and ATP, fix the transporter in a specific state: atractylosides (such as carboxyatractyloside; CAT) fixes the transporter in the “cytosolic” c-state thereby inducing swelling of mitochondria and apoptosis, and bongkrekic acid (BKA) fixes the transporter in the “matrix” m-state thereby suppressing induction of apoptosis [60]. LncP was not inhibited by CAT or BKA (Figure 6B). It was also not inhibited by the SH alkylating reagent N-ethylmaleimide (NEM; inhibitor of the phosphate, glutamate and ornithine carriers). In contrast, ATP transport catalyzed by LncP was effectively prevented by other reagents such as mersalyl (MER), p-hydroxymercurybenzoate (p-HMB) and HgCl2, which are organic mercurials, and by pyridoxal-5′-phosphate (PLP) and bathophenanthroline (BAT), which alone or in combination inhibit the activity of several mitochondrial carriers, although their mechanism of action is not known. Therefore, both the substrate specificity (Figure 6A) and the inhibitor sensitivity (Figure 6B) of LncP distinguish it biochemically from the ADP/ATP carrier.

To characterize further the transport properties of LncP, the kinetics of [^3^H]ATP and [^3^H]GTP uptake into proteoliposomes were compared either as uniport (in the absence of internal substrate) or as exchange (in the presence of internal ATP or GTP, respectively) (Figure 6C). Both the exchange and the uniport reactions of ATP and GTP uptake followed first-order kinetics, isotopic equilibrium being approached exponentially. The ratio of maximal substrate uptake by both reactions was 9.8 for ATP and 13.0 for GTP, in good agreement with the expected ratio of 10 from the intraliposomal concentrations at equilibrium (1 mM and 10 mM for uniport and exchange, respectively). The uniport mode of transport of reconstituted LncP was also investigated by measuring the efflux of [^3^H]ATP from pre-labeled proteoliposomes (Figure 6D) because this approach provides a more sensitive assay for unidirectional transport [61]. A significant efflux of ATP was observed in the absence of external substrate (filled circle) and a more rapid and extensive efflux occurred upon addition of ATP (open square) or phosphate (open triangle). Moreover, the ATP-induced efflux of radioactivity was prevented by the presence of the carrier inhibitors PLP and BAT (filled square). Similar results were obtained using GTP as substrate (data not shown). Thus, LncP was able to catalyze unidirectional transport of ATP and GTP and a fast exchange reaction of substrates.

## Discussion

**Figure 7:**
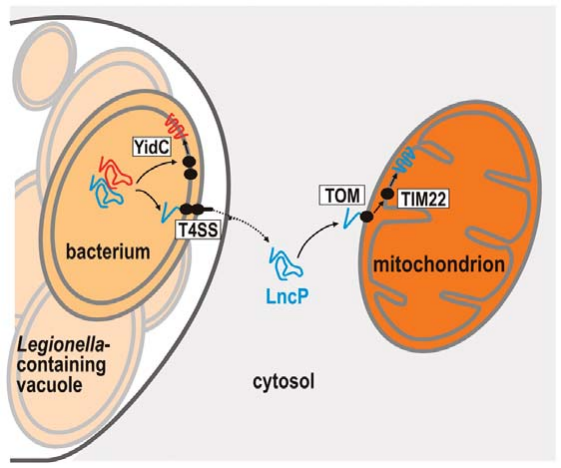
Transport of LncP across five membranes. Unlike regular bacterial inner membrane proteins with alpha-helical transmembrane segments (non-Dot/Icm effectors) (red), LncP (blue) avoids the YidC and SecYEG machinery in the bacterial inner membrane and is instead loaded into the T4SS for secretion across both the inner and outer bacterial membrane and across the vacuolar membrane. Similar to endogenous carrier proteins, LncP is then presumably recognized by Hsp70 and Hsp90 chaperones in the host cell cytosol and delivered to the TOM complex via interactions with the Tom70 receptor. The protein is then translocated across the outer mitochondrial membrane and interacts with the Tim9/10 chaperones in the intermembrane space to be assembled into the mitochondrial inner membrane by the TIM22 complex. There, the transport activity of LncP would impact on nucleotide homeostasis between the mitochondrial matrix and host cell cytosol.

Recently, 275 effectors of the Dot/Icm secretion system were described in the Philadelphia-1 strain of *L. pneumophila* [13]. This represents almost 10% of all open reading frames encoded in the *L. pneumophila* genome. Given that there is also diversity in the presence and range of effector genes among the different sequenced *L. pneumophila* genomes and even greater differences between *Legionella* species [22], the total Dot/Icm effector repertoire is likely to be much larger. Here we describe a new Dot/Icm effector from *L. pneumophila*, LncP, with sequence and functional similarity to eukaryotic mitochondrial carrier proteins. LncP was predicted to have six transmembrane domains, similar to eukaryotic MCF members. Remarkably, this highly hydrophobic protein crosses five biological membranes to reach its final destination in the mitochondrial inner membrane. Generally bacterial membrane proteins are assembled into the cytoplasmic membrane by YidC and SecYEG [62], reviewed in [63]. Chaperones for the Dot/Icm machinery, such as IcmS, IcmW and LvgA [64]–[66], must be in active competition with the bacterial YidC/SecYEG machinery to dictate which integral membrane proteins will be assembled into the bacterial inner membrane and which will be evacuated via the Dot/Icm T4SS. Therefore recognition of LncP by the Dot/Icm machinery presumably allows this hydrophobic protein to avoid assembly into the bacterial inner membrane by YidC/SecYEG (Figure 7). The mechanism by which this recognition occurs is unknown but probably involves detection of a C-terminal Dot/Icm secretion signal. Here we removed the C-terminal amino acids PTRKR from LncP but found that this had no effect on LncP translocation. Bioinformatic analysis of known Dot/Icm substrates has revealed a preference for short acidic or negatively charged amino acids in the C-terminal secretion signal [26], [53]. Recently, a glutamate rich region (E Block) was associated with the translocation signal of many Dot/Icm effectors [67]. The E Block motif was located in the C-terminal 30 amino acids of the effectors. LncP also contains a putative E Block motif in the C-terminus that may contain the signal for translocation (Figure 1A). However, the motif is predicted to lie within the most distal transmembrane domain of the carrier protein and likely contributes to correct protein folding and function. Hence, further investigation of the LncP secretion signal will require careful mutational analysis by amino acid substitution rather than deletion to dissect the bona fide secretion signal from the transmembrane domain.

The mechanism by which hydrophobic membrane proteins such as LncP can be accommodated in the translocase channel and assisted on the host cytoplasmic side of the *Legionella*-containing vacuole membrane without aggregating is unknown. When we analyzed the Dot/Icm effector repertoire of *L. pneumophila* 130b using two independent hidden Markov model approaches, HMMtop [68] and TMHMM v 2.0 [69], 71 effectors were predicted by both methods to have one or more transmembrane segments (Table S2). Thus the Dot/Icm T4SS has evolved to handle the export of proteins with significant hydrophobicity across at least three biological membranes.

Currently, mitochondrial localization of only one other Dot/Icm effector, LegS2, has been reported, although the precise mitochondrial compartment was not described. LegS2 has sphingosine-1-phosphate lyase activity and it is not yet clear if mitochondrial targeting plays any role in effector function [27]. Here we found that LncP was also targeted to mitochondria during infection of eukaryotic cells with *L. pneumophila* and assembled into the mitochondrial inner membrane, where the effector appeared to act as a unidirectional nucleotide transporter. Mitochondrial import required the TIM9:10 chaperones and hence the TIM22 machinery, according to classical mitochondrial protein transport mechanisms.

In the yeast model system, expression of LncP led to a dominant-negative phenotype. Although not lethal, the expression of LncP greatly slowed growth, particularly growth on minimal media. This dominant-negative phenotype depended on the activity of the endogenous MCF protein, Aac1. Whereas the yeast MCFs Aac2 and Aac3 are classic ADP/ATP carriers that regenerate cytoplasmic ATP levels (because ATP export can only be achieved with a concomitant import of ADP), a distinguishing feature of Aac1 is its propensity to export ATP from the mitochondrial matrix [57]. Thus, the dominant-negative effect seen in yeast is likely a cellular consequence of an imbalance of ADP/ATP transport across the mitochondrial inner membrane.

We also observed ATP transport activity for LncP in reconstituted liposomes. The kinetic parameters of ATP transport by LncP were comparable to those of genuine ATP carriers. There are two classes of transporters for ATP in the mitochondrial inner membrane: the carboxyatractyloside-inhibitable ADP/ATP carriers (Aac) and the ATP-Mg/Pi carriers (in humans named APC and in yeast Sal1). Studies in which the V_max_ of Aac has been measured in reconstituted liposomes (either as ATP/ATP or ADP/ADP exchange) using protein purified from mitochondria or after heterologous expression, obtained V_max_ values ranging from 360 and 1300 mmol/min/g protein [70]–[74]. Here we measured the V_max_ of ATP transport in LncP-reconstituted liposomes (measured as ATP/ATP exchange) as 926 mmol/min/g protein. This means that the ratio between the activity of LncP and the activity of genuine ADP/ATP carriers varied from 2.6 to 0.7. The K_m_ for ATP of genuine mitochondrial ADP/ATP carriers, measured in reconstituted liposomes, ranges between 9 and 120 µM, lower than the K_m_ of LncP for ATP (190 µM). However, the internal concentration of ATP in respiring mitochondria is sufficiently high to saturate both LncP and Aac. To date there is no other data available about the kinetic parameters of carboxyatractyloside-sensitive ADP/ATP carriers either purified from mitochondria or after heterologous expression. For the ATP-Mg/Pi carrier, only the human orthologs encoded by the SLC25A23 and SLC25A24 genes have been reconstituted into liposomes [75]. The V_max_ values of the ATP-Mg/Pi carriers (measured as ATP/ATP exchange) ranged from 65 to 523 mmol/min/g protein, lower than the V_max_ of LncP. The K_m_ values of human ATP-Mg/Pi carriers for ATP (0.3 mM) are 1.5-fold higher than the Km of LncP for ATP. In conclusion, the ATP transport activity of reconstituted LncP is at least as high as that of the known mitochondrial ATP transporters and is therefore compatible with the conclusion that LncP catalyzes ATP efflux from the mitochondria of infected cells.

Our reconstitution studies suggested LncP could evacuate ATP from the membrane lumen (matrix) by either uniport or an exchange reaction with substrates (e.g., phosphate). It is not yet clear how this assists *L. pneumophila* infection, however the high carriage of *lncP* in strains of *L. pneumophila* and *L. longbeachae* suggests that control over mitochondrial adenine nucleotide levels favours *Legionella* replication and survival. While fundamental studies show that elevated levels of cytosolic ATP primes cells to respond to apoptosis-inducing stimuli [76], [77], our preliminary experiments indicated that over-expression of LncP alone was insufficient to change the rate or extent of HeLa cell death induced by the exogenous trigger, staurosporine (Figure S2). Thus the contribution of LncP activity to *L. pneumophila* intracellular replication and persistence remains to be determined.

*L. longbeachae* and *L. pneumophila* share only some aspects of their life-cycle, and genome sequence analysis suggests that while these bacteria have a highly conserved Dot/Icm T4SS, they secrete quite different pools of effectors [46]. Despite this, *L. longbeachae* also harbors a strong homolog of LncP and two other putative MCF proteins. Further prokaryotic MCF sequences were found in another intracellular macrophage pathogen, *N. sennetsu*, which causes an infectious mononucleosis-like disease called sennetsu ehrlichiosis [44]. The presence of these exclusively eukaryotic proteins in bacteria is curious and suggests that the genes encoding the MCF proteins were acquired at some stage by lateral gene transfer from a eukaryotic host. MCF proteins are found in almost all species of eukaryotes [78], including protists that support the growth of *L. pneumophila*. Based on previous analysis and our own HMM search we found MCF proteins in all of *Acanthamoeba* (unpublished), *Dictyostelium discoideum* [31], [79] and *Naegleria gruberi* [80]. The association of bacterial MCF proteins with intracellular pathogens suggests the proteins could play similar roles in the pathogenesis of all these organisms. Further work on the biochemical function of the bacterial MCF members will aid our understanding of how bacteria modulate mitochondrial function during infection.

## Materials and Methods

### Sequence Analysis

The methodology for hidden Markov model analysis has been described previously [81]. A hidden Markov model tailored from 34 manually compiled mitochondrial carrier protein sequences was built and used to scan UniProt (Release 12.4, containing Swiss-Prot Release 54.4 and TrEMBL Release 37.4). The program HMMER 2.3.2 was used in all calculations [82], and the search results were extracted with programs prepared in-house. Homology modeling of the mitochondria carrier protein was performed with SwissModel [83] using the structure of bovine ANT (PDB ID 2C3E) as the template [47]. Sequences were aligned using ClustalX [84] and further edited in BioEdit (http://www.mbio.ncsu.edu/BioEdit/bioedit.html).

### Bacterial Strains and Culture Conditions

*L. pneumophila* strain 130b and derivatives were grown on buffered charcoal-yeast extract (BCYE) agar or in ACES [*N*-(2-acetamido)-2-aminoethanesulfonic acid]-buffered yeast extract broth at 37°C. *E. coli* strains were cultured aerobically in Luria broth (LB) or on LB agar. When required, antibiotics were used at the following final concentrations: ampicillin at 100 µg/ml; kanamycin at 100 µg/ml for *E. coli*, at 25 µg/ml for *L. pneumophila*; chloramphenicol at 12.5 µg/ml for *E. coli*, at 6 µg/ml for *L. pneumophila*.

### Yeast Culture and Cell Fractionation

*Saccharomyces cerevisiae* strain W303a was grown in rich medium or selective medium as previously described [85]. For ectopic expression of LncP in yeast, the complete *lncP* open reading frame was amplified by PCR from *L. pneumophila* 130b genomic DNA and cloned into p425MET25 and p416MET25 for complementation or GFP-LncP localization respectively. The individual carrier deletion**mutants (in a BY4741 background) were purchased from Open Biosystems. For the preparation of mitochondria yeast cultures were grown in rich medium containing lactate as a carbon source (YPlac media) at 25°C. Mitochondria were isolated by differential centrifugation as described previously [85], [86]. For the growth assays the cells were grown to a mid-logarithmic phase in a complete medium, diluted to OD_600_ = 0.2, spotted in a series of five-fold dilutions on the plates and incubated at 30°C for 3–6 days.

### Isolation of Yeast Mitochondria

For the isolation of wildtype and Tim10 depleted mitochondria the *Saccharomyces cerevisiae* strains W303, P*_MET3_Tim10* [87] were grown in synthetic glucose media [0.67% (w/v) yeast nitrogen, 2% (w/v) glucose, 0.01% (w/v) leucine, tryptophan, uracil, adenine and histidine at 30°C for 10 hours as a pre-culture. The pre-culture was diluted to *A_600_*_ nm_ = 0.2units/mL in media supplemented with 0.2 mM methionine then grown for 2 days to reach *A_600_*_ nm_ = 1.0 before harvesting and mitochondrial isolation by previously described methods [86].

### Synthesis of [^35^S]-labeled LncP

DNA encoding LncP was amplified and cloned into pSP73 (Promega) from genomic DNA isolated *Legionella pneumophila* (strain 130b). The oligonucleotides LncP-FW BamHI (GCGCGGATCCATGAAAGACAAAACAATA), and LncP-REV XhoI (GATCCTCGAGCTACCTGTTCCTTGTTGG) were used to amplify full length LncP DNA *In vitro* transcription was carried out as previously described [88]. Rabbit reticulocyte lysate was purchased from Promega and *in vitro* translation reactions were carried out for 30–60 minutes in the presence of [^35^S]- methionine/cysteine (MP Biomedicals) [88].

### *In vitro* Import Reactions

[^35^S]-Methionine/cysteine-labeled LncP or PiC were synthesized *in vitro* and were incubated with mitochondria (50 µg per lane) for the indicated time periods at 25°C in import buffer (0.6 M sorbitol, 50 mM Hepes (pH 7.4), 2 mM KPi (pH 7.4), 25 mM KCl, 10 mM MgCl_2_, 0.5 mM EDTA, 1 mM dithiothreitol, 4 mM ATP, and 2 mM NADH). Samples were treated with Proteinase K (40 µg/mL) in import buffer for 15 minutes on ice to remove un-imported material before addition of protease inhibitor (1 mM PMSF). The mitochondria were re-isolated by centrifugation at 10,000 g and this was followed by either protein separation under denaturing gel electrophoresis (SDS) or protein complexes separated by Blue Native electrophoresis [89]. For Proteinase K shaving or mitochondrial membrane potential dissipation conditions, mitochondria were treated with 40 µg/ml Proteinase K or AVO mix (8 µM antimycin A, 1 µM valinomycin, and 20 µM oligomycin) respectively. For preparation of mitoplasts (mitochondria with ruptured outer mitochondrial membrane), post-import mitochondria were subjected to osmotic shock by resuspension in 20 mM Hepes/KOH, pH 7.4 with and without Proteinase K where indicated.

### Sodium Carbonate Extractions

After the completion of *in vitro* import reactions for 8 minutes at 25°C, mitochondria were re-isolated by centrifugation at 10,000 g and resuspended in 200 µL of 100 mM sodium carbonate which was adjusted to pH 11.5 and left on ice for 30 minutes with gentle mixing every 5 to 10 minutes. A membrane pellet was then separated from a supernatant by ultra-centrifugation at 100,000g for 30 minutes at 4°C. The pellet was resuspended in 200 µL of 100 mM sodium carbonate and both the pellet and supernatant were then subjected to Trichloroacetic acid precipitation. Each experiment was conducted in duplicate with one set of pellet and supernatant samples recombined to make the “total” sample. This was repeated 5 times in order to assess statistical significance. LncP protein level was quantified by densitometry of phosphorimages using the Image Quant software.

### Reconstitution of LncP into Liposomes and Transport Measurements

Expression of recombinant LncP is detailed in the supporting methods (Protocol S1). The recombinant, purified LncP was reconstituted into liposomes by cyclic removal of the detergent with a hydrophobic column of Amberlite beads (Fluka) [61]. The composition of the initial mixture used for reconstitution was 35 µl of purified LncP (15 µg of protein), 70 µl of 10% Triton X-114, 100 µl of 10% phospholipids in the form of sonicated liposomes, 10 mM ATP (except where otherwise indicated), 10 mM PIPES (pH 7.0), 0.42 mg of the mitochondrial lipid cardiolipin (Sigma) and water to a final volume of 700 µl. After vortexing, this mixture was recycled 13 times through the Amberlite column (3.5×0.5 cm) pre-equilibrated with a buffer containing 10 mM PIPES pH 7.0. All steps were performed at 4°C, except for the passages through Amberlite, which were carried out at room temperature.

External substrate was removed from proteoliposomes on Sephadex G-75 columns pre-equilibrated with 50 mM NaCl and 10 mM PIPES at pH 7.0 (buffer A) and 4°C. The eluted proteoliposomes were distributed in reaction vessels and used for transport measurements by the inhibitor-stop method [61]. Transport at 25°C was started by adding [^3^H]ATP (Perkin Elmer) or [^3^H]GTP (American Radiolabeled Chemicals) to proteoliposomes and terminated by addition of 20 mM pyridoxal-5′-phosphate and 20 mM bathophenanthroline. In controls, the inhibitors were added at the beginning together with the radioactive substrate. Finally, the external radioactivity was removed from each sample of proteoliposomes by a Sephadex G-75 column; the proteoliposomes were eluted with buffer A and their radioactivity was measured. The experimental values were corrected by subtracting control values. The initial transport rate was calculated from the radioactivity taken up by proteoliposomes after 2 min (in the initial linear range of substrate uptake). For efflux measurements, proteoliposomes containing 2 mM ATP or GTP were labeled with 10 µM [^3^H]ATP or [^3^H]GTP, respectively, by carrier-mediated exchange equilibration [61]. After 50 min, the external radioactivity was removed by passing the proteoliposomes through Sephadex G-75 pre-equilibrated with buffer A. Efflux was started by adding unlabeled external substrate or buffer A alone to aliquots of proteoliposomes and terminated by adding the inhibitors indicated above.

### Disruption of LncP in *L. pneumophila* 130b

An insertional mutation in LncP was created via homologous recombination. A ∼1 kb fragment encompassing LncP was amplified by PCR from *L. pneumophila* 130b genomic DNA using the oligonucleotide primers, 5′- caacggatcctatttcatttgtagtcccttg -3′ and 5′- tcctgtcgacctgaaatattttcatggaaac -3′. The resulting product was cloned into the *BamH*I and *Sal*I sites of pPCRScript and a kanamycin resistance gene from Tn*5* was introduced into the native *Pst*I site of LncP. The construct was introduced into *L. pneumophila* 130b via natural transformation, as described previously [90]. Kanamycin resistant clones were assessed by PCR analysis and ampicillin sensitivity to detect replacement of *lncP* with *lncP::km* and the loss of pCR-Script. Two independent *L. pneumophila lncP::km* clones, LncP*-3* and LncP*-4,* were chosen for further analysis in host cell replication assays.

### Macrophage and HeLa Cell Infection and Anti-HA Immunofluorescence

The human monocytic cell line, THP-1 was maintained in RPMI 1640 supplemented with 10% fetal bovine serum in 5% CO_2_ at 37°C. The cells were prepared for infection with stationary-phase *L. pneumophila* as previously described [91]. THP-1 cells were infected at a multiplicity of infection (MOI) of 5 cells for 2 h in 5% CO_2_ at 37°C. Cells were then treated with 100 µg/ml gentamicin for 1 h to kill extracellular bacteria and washed with PBS before being lysed with 0.01% digitonin. Serial dilutions of the inoculum and bacteria recovered from lysed cells were plated on BCYE agar and results were expressed as the percentage of the inoculum that resisted killing by gentamicin (mean ± standard deviation of at least 3 independent experiments).

Immortalized macrophages from wild type C57BL/6 mice [92] were seeded at 2×10^5^ per coverslip 16 h prior infection. The B6 macrophages were a gift from Dr Ashley Mansell (Monash Institute of Medical Research). Cells were maintained in DMEM supplemented with 10% FCS, 2 mM glutamine, 100 U penicillin/ml and 100 µg streptomycin/ml. Immediately prior to infection, macrophages were washed and the medium replaced with DMEM supplemented with 1 mM IPTG and lacking antibiotics. Macrophages were infected for 30 min, 1 h, 2 h, 3 h or 5 h with derivatives of *L. pneumophila* 130b at a multiplicity of infection of 50. Bacterial strains for infection were grown overnight in ACES broth supplemented with antibiotics where appropriate and 1 mM IPTG. HeLa cells were infected using an identical protocol. Following the infection period, cells were washed once with fresh tissue culture medium and incubated with 500 nM Mitotracker® Red FM (Invitrogen) for 30 min at 37°C and 5% CO_2_. Labelled cells were then fixed in 4% paraformaldehyde-PBS for 20 min and permeabilized with 0.1% TritonX-100-PBS for 20 min. Cells were incubated for 60 min in staining solution containing 0.2% BSA, 1∶50 dilution of anti-HA.11 monoclonal antibody (Covance) and 1∶75 dilution of rabbit raised anti-*Legionella pneumophila* antibody (Acris). The bound primary antibodies were detected using 1∶1000 dilution of Alexa Fluor 405-conjugated anti-rabbit antibody and Alexa Fluor 488-conjugated anti-mouse antibody (Invitrogen) respectively. Coverslips were mounted onto glass slides with Dako Fluorescent Mounting Medium (Dako). Immunofluorescence images were acquired using a confocal laser scanning microscope (Leica TCS SP2 confocal imaging system) with a 100x/1.4 NA HCX PL APO CS oil immersion objective.

### Cya-LncP and 4HA Gene Fusions and Intracellular cAMP Assays

Adenylate cyclase (Cya) fusions with RalF and LncP were generated as described previously [51]. Details are provided in the supporting methods (Protocol S1). Hemagluttinin (HA) fusions with LncP were generated as described in the supporting methods (Protocol S1).

### Infection of *A. castellanii* with *L. pneumophila*

*A. castellanii* ATCC 50739 was cultured in PYG 712 medium at 20°C for 72 h prior to harvesting for *L. pneumophila* infection. *A. castellanii* cells were washed once with A.c. buffer (0.1% trisodium citrate, 0.4 mM CaCl_2_, 2,5 mM KH_2_PO_4_, 4 mM MgSO_4_, 2.5 mM Na_2_HPO_4_, 0.005 mM ferric pyrophosphate) and seeded into 24-well tissue culture trays (Sarstedt, Leicestershire, United Kingdom) at a density of 10^5^ cells/well. Stationary-phase *L. pneumophila* was added at an MOI of 0.01 and incubated at 37°C. At set time points, entire co-culture volumes were collected and plated onto BCYE agar to count colony-forming units of *L. pneumophila*.

### LncP Purification

Proteins were analyzed by SDS-PAGE or by Blue Native (BN)-PAGE (Figure S3) as previously described [85]. N-terminal sequencing was carried out as described previously [93]. Purified LncP was quantified by laser densitometry of stained samples, using carbonic anhydrase as the protein standard [93]. Protein incorporation into liposomes was measured as described [93] and varied between 20-30% of the protein added to the reconstitution mixture.

## Supporting Information

Figure S1 **Localization of 4HA-LncP in macrophages and HeLa cells.** (A) Macrophages were infected with *L. pneumophila* (either wild type 130b or the Δ*dotA* mutant) expressing 4HA-LncP. Bacteria were visualized using anti-*Legionella* antibodies (blue) 4HA-LncP was visualized with antibodies to HA (green). Prior to fixation, cell were stained with MitoTracker Red. Cells were viewed by confocal microscopy under a 100× objective. The merge shows the mitochondrial localization of 4HA-LncP. White scale bars represent 10 µm (B) HeLa cells infected with *L. pneumophila* (either wild type 130b or the Δ*dotA* mutant) expressing 4HA-LncP were analyzed as above.
(TIF)

Figure S2 **LncP is targeted to mitochondria, but does not impact on apoptosis induced by staurosporine treatment.** (A) LncP-EGFP was expressed in HeLa cells. Cells were immunostained with antibodies against GFP (green) and active caspase-3 (red). Hoechst 33342 was used as a counterstain to indicate nucleus (blue). The panel at the right shows the cells treated with staurosporine for 5–6 hours. The left panel shows the cells without staurosporine treatment. Scale bar: 10 µm. The Table documents the analysis by cell counting. Total cells were counted based on nucleus staining. LncP-EGFP expressing cells were counted based on green color while cells with active caspase-3 were counted based on red color. (B) HeLa cells were transfected with LncP-EGFP (green) and then stained with tetramethylrhodamine methyl ester (TMRM) (red). The right panel shows the cells treated with vehicle DMSO for 5 hours while the left panel is the cells without treatment. Scale bar: 50 µm (C) HeLa cells transfected with LncP-EGFP (green) were stained with TMRM (red) and then treated with staurosporine for up to 3 hours. The panel at the right shows fluorescence images while the left panel shows bright field images. Scale bar: 50 µm.
(TIF)

Figure S3 **Recombinant expression and purification of LncP.** Proteins were separated by SDS-PAGE and stained with Coomassie Blue. Markers in left-hand column (bovine serum albumin, carbonic anhydrase, and cytochrome *c*); lanes 1–4, *Escherichia coli* C0214 (DE3) containing the expression vector without (lanes 1 and 3) and with (lanes 2 and 4) the coding sequence of LncP. Samples were taken at the time of induction (lanes 1 and 2) and 5 h later (lanes 3 and 4). The same number of bacteria was analyzed in each sample. Lane 5, purified LncP protein (5 µg) purified from *E. coli* shown in lane 4. The identity of the purified protein was confirmed by N-terminal sequencing. Approximately 55mg of purified protein per liter of culture were obtained.
(TIF)

Protocol S1 **Supporting methods.**
(DOC)

Table S1 **Prevalence of** ***lncP*** **among strains of** ***L. pneumophila*** **.** A range of *L. pneumophila* strains were tested for carriage of *lncP* by Southern hybridisation as described previously [90]. A digoxigenin (DIG)-labelled probe was generated by PCR amplification according to the manufacturer's instructions (Roche) with the primer pair 5′- caacggatcctatttcatttgtagtcccttg -3′ and 5′- tcctgtcgacctgaaatattttcatggaaac -3′ using *L. pneumophila* 130b genomic DNA as a template [45].
(DOC)

Table S2 **Predicted membrane proteins in the** ***L. pneumophila*** **Dot/Icm effector repertoire.** Two hundred and seventy-five proteins encoded in the genome of *L. pneumophila* strain Philadelphia were identified as effector proteins in a recent high-throughput study [13]. Here the protein sequences of these effectors as well effectors unique to *L. pneumophila* strain 130b were analyzed with HMMtop [68] and TMHMM v 2.0 [69] to predict transmembrane segments. The two predictors have independent means of assessing hydrophobicity and other characteristics of alpha-helical transmembrane segments, yet concordant predictions are seen for most proteins.
(DOC)
