# Correction: *Legionella pneumophila* Secretes a Mitochondrial Carrier Protein during Infection

**DOI:** 10.1371/annotation/ee7c807b-032c-4d1f-b5ac-0f6620a2ef24

**Published:** 2012-01-20

**Authors:** Pavel Dolezal, Margareta Aili, Janette Tong, Jhih-Hang Jiang, Carlo M. T. Marobbio, Sau fung Lee, Ralf Schuelein, Simon Belluzzo, Eva Binova, Aurelie Mousnier, Gad Frankel, Giulia Giannuzzi, Ferdinando Palmieri, Kipros Gabriel, Thomas Naderer, Elizabeth L. Hartland, Trevor Lithgow

An initial is missing in the fifth author's name. The correct name is: Carlo MT Marobbio. The correct citation is: Dolezal P, Aili M, Tong J, Jiang J-H, Marobbio CMT, et al. (2012) Legionella pneumophila Secretes a Mitochondrial Carrier Protein during Infection. PLoS Pathog 8(1): e1002459. doi:10.1371/journal.ppat.1002459

