# Correction: *Legionella pneumophila* Secretes a Mitochondrial Carrier Protein during Infection

**DOI:** 10.1371/annotation/5039541e-b48a-4cfc-84b1-21566e311a62

**Published:** 2012-01-20

**Authors:** Pavel Dolezal, Margareta Aili, Janette Tong, Jhih-Hang Jiang, Carlo M. T. Marobbio, Sau fung Lee, Ralf Schuelein, Simon Belluzzo, Eva Binova, Aurelie Mousnier, Gad Frankel, Giulia Giannuzzi, Ferdinando Palmieri, Kipros Gabriel, Thomas Naderer, Elizabeth L. Hartland, Trevor Lithgow

An initial is missing in the fifth author's name. The correct name is: Carlo MT Marobbio. The updated Author Contributions are: "Conceived and designed the experiments: PD MA JT JHJ CMTM EB FP KG GG ELH TL. Performed the experiments: PD MA SFL RS JT JHJ CMTM EB GG. Analyzed the data: PD SB TN JT JHJ CMTM EB FP KG GG ELH TL. Contributed reagents/materials/analysis tools: AM GF. Wrote the paper: PD ELH TL FP."

