# Supplementary figures and images for: Legionella pneumophila Secretes a Mitochondrial Carrier Protein during Infection
Source: PLoS Pathog. 2012 Jan 5;8(1):e1002459. doi: 10.1371/journal.ppat.1002459 (PMC3252375; doi:10.1371/journal.ppat.1002459)

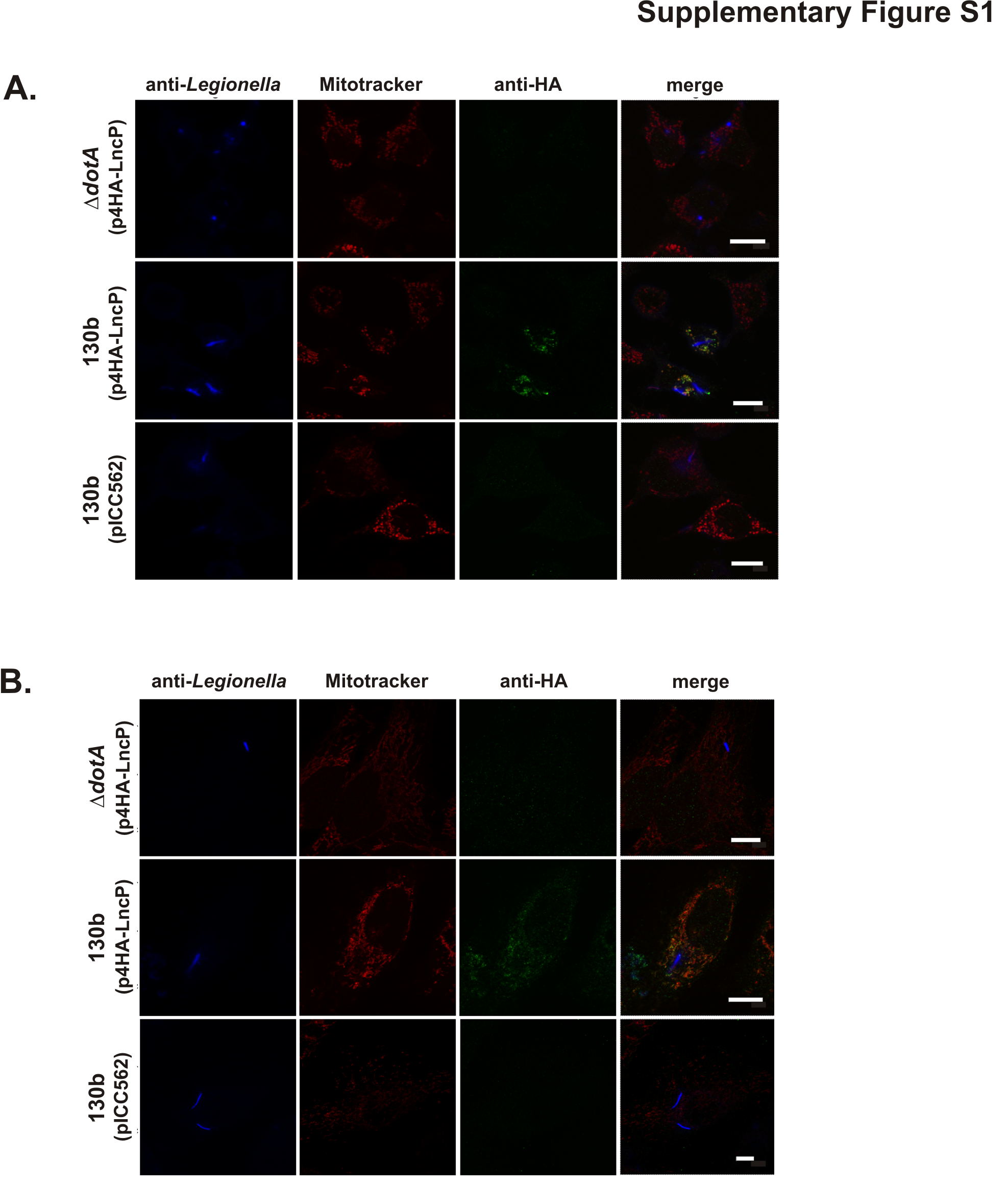

Supplement: Figure S1 — Localization of 4HA-LncP in macrophages and HeLa cells. (A) Macrophages were infected with L. pneumophila (either wild type 130b or the ΔdotA mutant) expressing 4HA-LncP. Bacteria were visualized using anti-Legionella antibodies (blue) 4HA-LncP was visualized with antibodies to HA (green). Prior to fixation, cell were stained with MitoTracker Red. Cells were viewed by confocal microscopy under a 100× objective. The merge shows the mitochondrial localization of 4HA-LncP. White scale bars represent 10 µm (B) HeLa cells infected with L. pneumophila (either wild type 130b or the ΔdotA mutant) expressing 4HA-LncP were analyzed as above. (TIF) [file ppat.1002459.s001.tif]

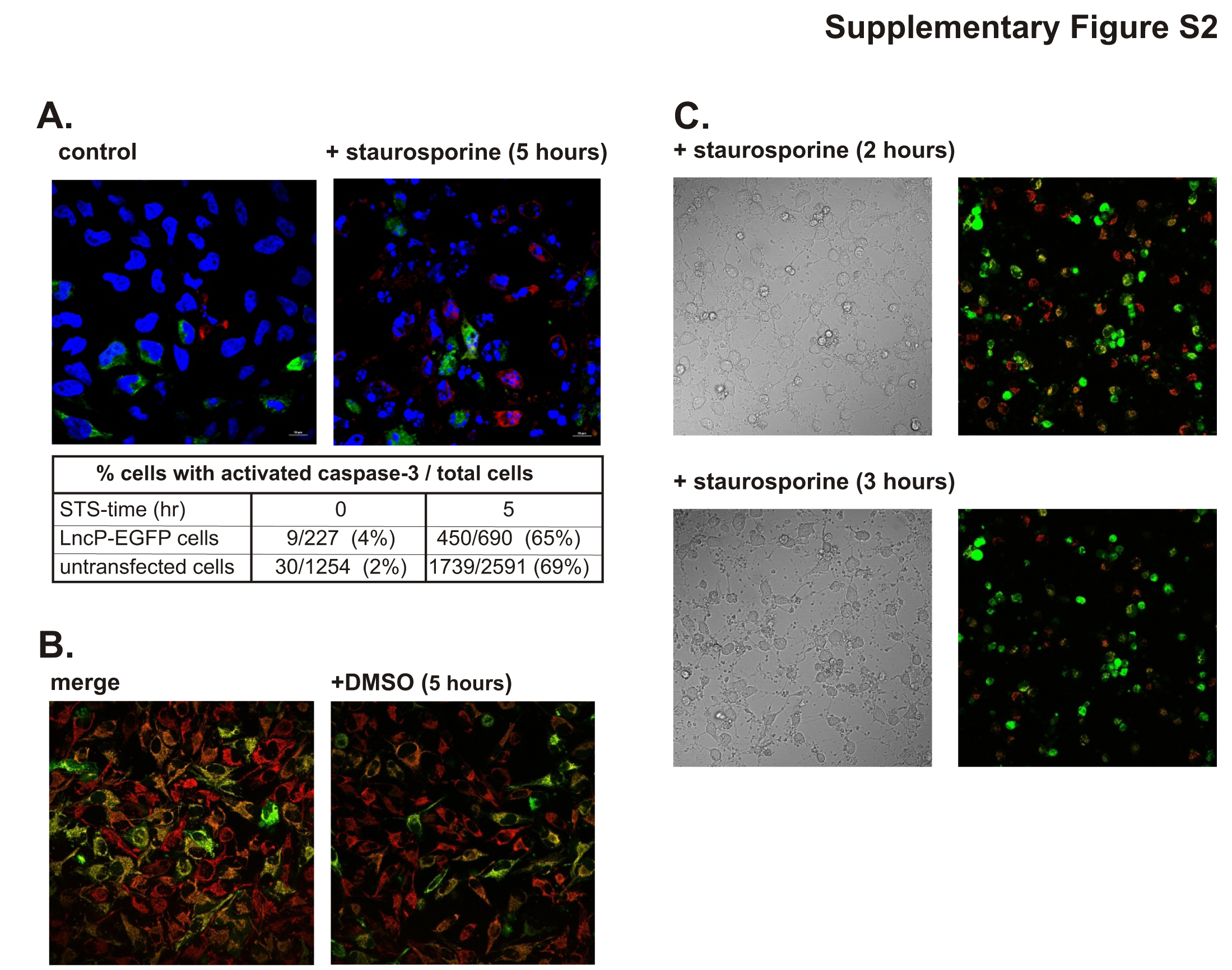

Supplement: Figure S2 — LncP is targeted to mitochondria, but does not impact on apoptosis induced by staurosporine treatment. (A) LncP-EGFP was expressed in HeLa cells. Cells were immunostained with antibodies against GFP (green) and active caspase-3 (red). Hoechst 33342 was used as a counterstain to indicate nucleus (blue). The panel at the right shows the cells treated with staurosporine for 5–6 hours. The left panel shows the cells without staurosporine treatment. Scale bar: 10 µm. The Table documents the analysis by cell counting. Total cells were counted based on nucleus staining. LncP-EGFP expressing cells were counted based on green color while cells with active caspase-3 were counted based on red color. (B) HeLa cells were transfected with LncP-EGFP (green) and then stained with tetramethylrhodamine methyl ester (TMRM) (red). The right panel shows the cells treated with vehicle DMSO for 5 hours while the left panel is the cells without treatment. Scale bar: 50 µm (C) HeLa cells transfected with LncP-EGFP (green) were stained with TMRM (red) and then treated with staurosporine for up to 3 hours. The panel at the right shows fluorescence images while the left panel shows bright field images. Scale bar: 50 µm. (TIF) [file ppat.1002459.s002.tif]

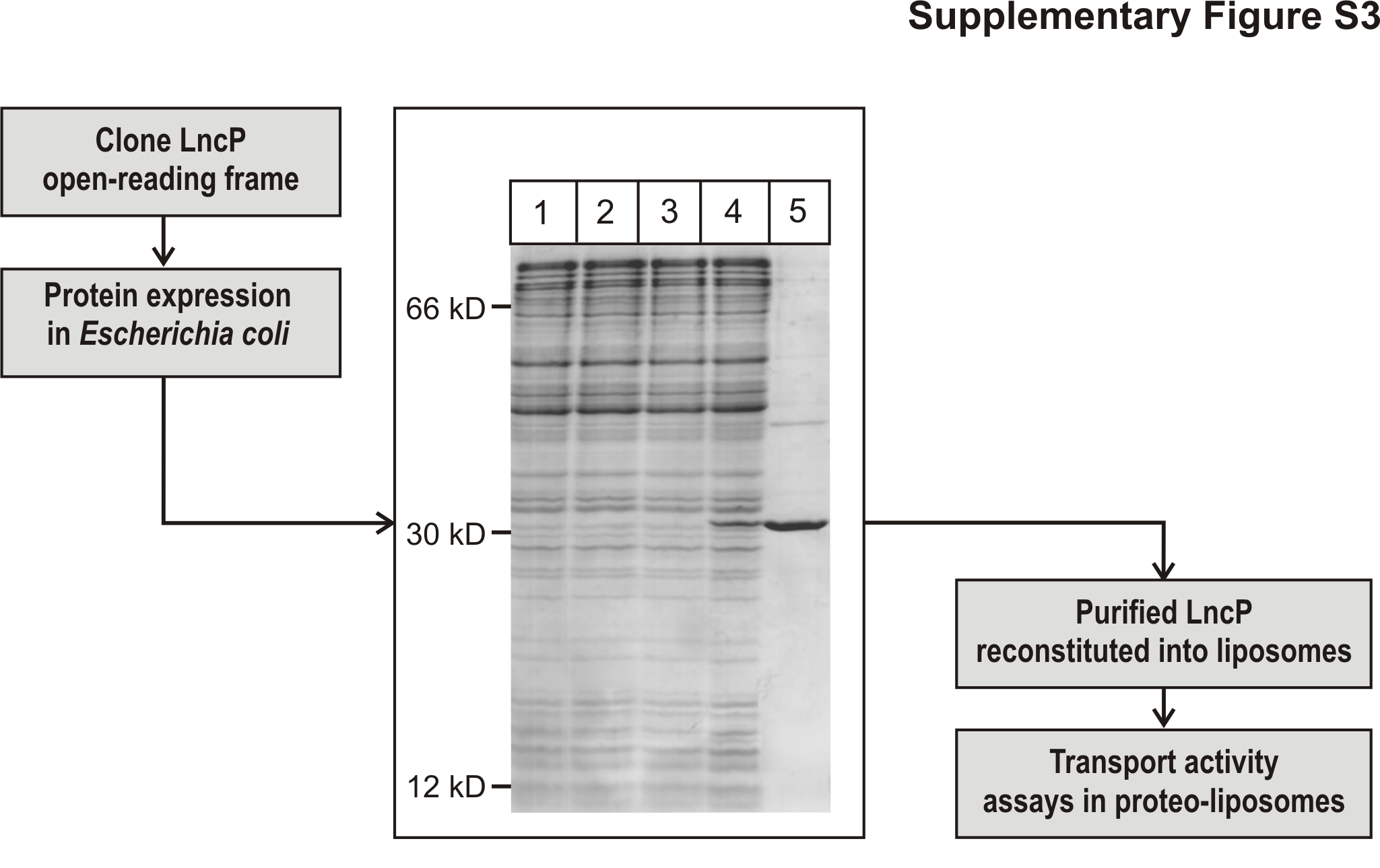

Supplement: Figure S3 — Recombinant expression and purification of LncP. Proteins were separated by SDS-PAGE and stained with Coomassie Blue. Markers in left-hand column (bovine serum albumin, carbonic anhydrase, and cytochrome c); lanes 1–4, Escherichia coli C0214 (DE3) containing the expression vector without (lanes 1 and 3) and with (lanes 2 and 4) the coding sequence of LncP. Samples were taken at the time of induction (lanes 1 and 2) and 5 h later (lanes 3 and 4). The same number of bacteria was analyzed in each sample. Lane 5, purified LncP protein (5 µg) purified from E. coli shown in lane 4. The identity of the purified protein was confirmed by N-terminal sequencing. Approximately 55mg of purified protein per liter of culture were obtained. (TIF) [file ppat.1002459.s003.tif]
