# Supplementary material for: Legionella pneumophila Secretes a Mitochondrial Carrier Protein during Infection
Source: PLoS Pathog. 2012 Jan 5;8(1):e1002459. doi: 10.1371/journal.ppat.1002459 (PMC3252375; doi:10.1371/journal.ppat.1002459)
Supplement: Protocol S1 — Supporting methods. (DOC) [file ppat.1002459.s004.doc]

**SUPPORTING METHODS**

**Expression and purification of recombinant LncP**

The coding sequence for LncP was amplified by PCR from genomic DNA with oligonucleotide primers carrying *Nde*I and *Eco*RI sites, and cloned into the pMW7 expression vector. To optimize the expression of LncP in *E. coli* CO214(DE3), as described below, silent modifications were made to the sequence of LncP by substituting the third base of codons 5, 6, 7 and 9 with C, C, G and G, respectively. Expression of LncP in inclusion bodies was accomplished as described previously [1] except that the host cells were *E. coli* CO214(DE3) [2]. Inclusion bodies were purified on a sucrose density gradient [1], washed at 4°C with TE buffer (10 mM Tris-HCl, 1 mM EDTA pH 7.0), then twice with a buffer containing Triton X-114 (3%, w/v), 20 mM Na_2_SO_4_, 1 mM EDTA and 10 mM PIPES pH 7.0, and twice again with TE buffer. LncP was solubilized in 1.8% sarkosyl (w/v) (Fig. S3).

**Cya-LncP gene fusions and intracellular cAMP assays**

Adenylate cyclase (Cya) fusions with RalF and LncP were generated as described previously [3,4]. Briefly, *ralF* and LncP were amplified using the primer pairs 5’-aggcggccgcaaatgcatccagagattg-3’ and 5’-aggcggccgcttatttcttataactcgatc-3’ (*ralF*) and 5’-aagagcggccgctaatgaaagacaaaacaataccc-3’ and 5’- aaaggcggccgcagggggcgttatgaatttac -3’ (LncP). A truncated form of LncP lacking the last 5 amino acids was generated with the primer pair 5’-aagagcggccgctaatgaaagacaaaacaataccc-3’ and 5’-cgctgcagttaccttccataagcttc-3’. The resulting products were cloned into the *Not*I or *Not*I/*Pst*I sites of pEC34 (pCya) to generate a translational fusion with the C-terminus of Cya [3].

Translocation assay was performed as described [4]. Briefly, THP-1 cells were seeded at a density of 5x10^5^ per well and chemically differentiated using Probol Myristate Acetate (PMA) at final concentration 50 ng/ml and incubated for 2 days at 37°C and 5% CO_2_ prior to infection. Bacteria expressing hybrid Cya-proteins were grown over night in ACES containing appropriate antibiotics and 1 mM IPTG. Bacteria were added to THP-1 monolayers (MOI of 10) and centrifuged at 200 *g* for 5 min to initiate contact and synchronize infection. After 1 h incubation at 37°C and 5% CO_2_, the monolayers were washed with cold PBS and lysed in 50 mM HCl + 0.1% v/v Triton X-100 before boiling for 5 min. The cell lysates were neutralized with 30 mM NaOH and centrifuged at 600 *g* for 5 min. Levels of cAMP in cell lysates were determined using the cAMP BioTrak enzyme immunoassay (EIA) system (Amersham Biosciences).

**Expression of 4HA-LncP by *L. pneumophila***

The plasmid, pICC562 (pMMB207C-HAx4) was constructed to allow effectors to be cloned into available restriction sites (KpnI/SmaI/BamHI/XbaI/HindIII sites) as translational fusions with 4 HA epitope tags. Effector expression may be induced upon induction with 1.0 mM IPTG. The vector was generated by hybridizing the oligonucleotide pair 5’-tatgtacccatacgatgttccagattacgcttacccatacgatgttccag attacgcttacccatacgatgttccagattacgcttacccatacgatgttccagattacgctggcgcgggtac-3’ and 5’-ccgcgccagcgtaatctggaacatcgtatgggtaagcgtaatctggaacatcgtatgggtaagcgtaatctggaacatcgtatgggtaagcgtaatctggaacatcgtatgggtaca-3’. The resulting product was cloned in place of the *blaM* gene of the pXDC61 plasmid into the *Nde*I/*Kpn*I sites [5]. p4HA-LncP was generated by cloning the gene encoding LncP into the *Sma*I and *BamH*I sites of pICC562. LncP was amplified from 130b genomic DNA using the primer pair amplified using the oligonucleotide primers, 5’-gcgcccggggaatgaaagacaaaacaatacc-3’ and 5’-ggtggatccaatttactccctgttcc-3’. The resulting recombinant plasmids were verified by DNA sequencing using the oligonucleotide primers 5’-ttgacaattaatcatcggc-3’ and 5’-aggcaaattctgttttatc-3’. pHA-LncP was transformed into *L. pneumophila* 130b and the *dotA* mutant as described above. Expression of 4HA-LncP in each background was checked by Western blot using anti-HA antibodies (monoclonal antibody HA.11, Covance, Emeryville, CA) diluted 1:2,000.

**Culture of HeLa cells and fluorescence microscopy**

HeLa cells were cultured in Dulbecco’s Modified Eagle Medium (DMEM) containing 10% fetal bovine serum and incubated in an atmosphere containing 5% CO_2_ at 37°C. LncP was cloned into pEGFP-N1 (Clonetech). LncP-EGFP plasmid was introduced into HeLa cells with Lipofectamine 2000 (Invitrogen) following manufacture’s protocol. Cells were then incubated with 4 μM staurosporine (SIGMA) for the time periods indicated in figure legends. For live cell imaging, cells were stained with tetramethylrhodamine methyl ester (TMRM, Invitrogen) in 37°C for 15 min and then washed twice with phenol red-free medium before adding staurosporine. For active caspase 3 detection, cells were fixed with 4% formaldehyde and then permeabilized with 0.1% Triton X-100. Rabbit polyclonal antibody against active caspase 3 and mouse monoclonal antibody against GFP were then used for immuno-staining following manufacture’s guidelines (AbCam). Goat anti rabbit 594 and goat anti mouse 488 antibodies were used for secondary antibodies following manufacture’s protocol (Invitrogen). Hoechst 33342 (Invitrogen) was used with 0.1μg/ml for counterstaining. Confocal fluorescence microscopy was performed using Nikon C1 Inverted microscope. Images were acquired with 60 and 100 times oil immersion objective (1.4 numerical aperture) using Nikon Elements software (Nikon Corporation, Japan).

**REFERENCES**

1. Fiermonte G, Walker JE, Palmieri F (1993) Abundant bacterial expression and reconstitution of an intrinsic membrane-transport protein from bovine mitochondria. Biochem J 294 ( Pt 1): 293-299.

2. Marobbio CM, Agrimi G, Lasorsa FM, Palmieri F (2003) Identification and functional reconstitution of yeast mitochondrial carrier for S-adenosylmethionine. EMBO J 22: 5975-5982.

3. Nagai H, Cambronne ED, Kagan JC, Amor JC, Kahn RA, et al. (2005) A C-terminal translocation signal required for Dot/Icm-dependent delivery of the *Legionella* RalF protein to host cells. Proc Natl Acad Sci U S A 102: 826-831.

4. Cambronne ED, Roy CR (2007) The *Legionella pneumophila* IcmSW complex interacts with multiple Dot/Icm effectors to facilitate type IV translocation. PLoS Pathog 3: e188.

5. de Felipe KS, Glover RT, Charpentier X, Anderson OR, Reyes M, et al. (2008) *Legionella* eukaryotic-like type IV substrates interfere with organelle trafficking. PLoS Pathog 4: e1000117.
