# Supplementary material for: Legionella pneumophila Secretes a Mitochondrial Carrier Protein during Infection
Source: PLoS Pathog. 2012 Jan 5;8(1):e1002459. doi: 10.1371/journal.ppat.1002459 (PMC3252375; doi:10.1371/journal.ppat.1002459)
Supplement: Table S1 — Prevalence of lncP among strains of L. pneumophila . A range of L. pneumophila strains were tested for carriage of lncP by Southern hybridisation as described previously [90]. A digoxigenin (DIG)-labelled probe was generated by PCR amplification according to the manufacturer's instructions (Roche) with the primer pair 5′- caacggatcctatttcatttgtagtcccttg -3′ and 5′- tcctgtcgacctgaaatattttcatggaaac -3′ using L. pneumophila 130b genomic DNA as a template [45]. (DOC) [file ppat.1002459.s005.doc]

**Table S1. Prevalence of *lncP* among strains of *L. pneumophila*.**

| *L. pneumophila* strain | Serogroup and source | *lncP* | Reference |
| --- | --- | --- | --- |
| Philadelphia-1 | O1; clinical isolate | − | [1] |
| Paris | O1; clinical isolate | + | [2] |
| Corby | O1; clinical isolate | + | [3] |
| 130b | O1; clinical isolate | + | [4] |
| 02/40 | O1; clinical isolate | + | [5] |
| 02/41 | O1; Environmental isolate | + | [5] |
| 03/41 | O1; environmental isolate | + | [5] |
| 03/42 | O1; environmental isolate | + | [5] |
| 03/43 | O1; environmental isolate | + | [5] |
| 03/45 | O1; environmental isolate | + | [5] |
| 03/46 | O1; clinical isolate | + | [5] |
| 03/47 | O1; clinical isolate | + | [5] |
| 03/48 | O1; clinical isolate | + | [5] |
| 03/49 | O1; clinical isolate | + | [5] |
| 03/50 | O1; clinical isolate | − | [5] |
| 03/53 | O3; environmental isolate | − | [5] |
| 03/54 | O3; environmental isolate | + | [5] |
| 03/55 | O4; environmental isolate | + | [5] |
| 03/56 | O4; environmental isolate | + | [5] |
| 03/57 | O5; environmental isolate | + | [5] |
| 03/59 | O6; environmental isolate | + | [5] |
| 03/60 | O6; environmental isolate | − | [5] |
| 03/61 | O7; environmental isolate | + | [5] |
| 03/63 | O8; environmental isolate | − | [5] |
| 03/64 | O8; environmental isolate | + | [5] |
| CT1 | O1; environmental isolate | + | [6] |
| PN1 | O1; environmental isolate | + | [6] |
| PN2 | O1; environmental isolate | + | [6] |
| B6 | O1; environmental isolate | + | [6] |
| CT3C | O1; environmental isolate | + | [6] |
| C102 | 2-14; environmental isolate | + | [6] |
| C42 | 2-14; environmental isolate | + | [6] |
| C7 | 2-14; environmental isolate | − | [6] |
| C1 | 2-14; environmental isolate | + | [6] |
| CT67 | 2-14; environmental isolate | − | [6] |
| CT4b | 2-14; environmental isolate | − | [6] |
| **Total** |  | **28/36 (77%)** |  |

**References**
