# Supplementary material for: Legionella pneumophila Secretes a Mitochondrial Carrier Protein during Infection
Source: PLoS Pathog. 2012 Jan 5;8(1):e1002459. doi: 10.1371/journal.ppat.1002459 (PMC3252375; doi:10.1371/journal.ppat.1002459)
Supplement: Table S2 — Predicted membrane proteins in the L. pneumophila Dot/Icm effector repertoire. Two hundred and seventy-five proteins encoded in the genome of L. pneumophila strain Philadelphia were identified as effector proteins in a recent high-throughput study [13]. Here the protein sequences of these effectors as well effectors unique to L. pneumophila strain 130b were analyzed with HMMtop [68] and TMHMM v 2.0 [69] to predict transmembrane segments. The two predictors have independent means of assessing hydrophobicity and other characteristics of alpha-helical transmembrane segments, yet concordant predictions are seen for most proteins. (DOC) [file ppat.1002459.s006.doc]

**Table S2. Predicted membrane proteins in the *L. pneumophila* Dot/Icm effector repertoire.**

| **Gene name** | **HMMtop** | **TMHMM** |
| --- | --- | --- |
| [lpg1661](http://www.ncbi.nlm.nih.gov/protein/Q5ZUY1) | 10 | 10 |
| [lpg2552](http://www.ncbi.nlm.nih.gov/protein/Q5ZSG5) | 9 | 8 |
| [MavN](http://www.ncbi.nlm.nih.gov/protein/Q5ZRR5) | 8 | 7 |
| [lpg2888](http://www.ncbi.nlm.nih.gov/protein/Q5ZRJ3) | 7 | 5 |
| [lpg0716](http://www.ncbi.nlm.nih.gov/protein/Q5ZXL5) | 7 | 4 |
| [RavC](http://www.ncbi.nlm.nih.gov/protein/Q5ZZ41) | 6 | 5 |
| [LncP](http://www.ncbi.nlm.nih.gov/protein/307611796) | 6 | 3 |
| [Ceg2](http://www.ncbi.nlm.nih.gov/protein/Q5ZZF1) | 5 | 6 |
| [MavP](http://www.ncbi.nlm.nih.gov/protein/Q5ZRJ7) | 5 | 5 |
| [SidA](http://www.ncbi.nlm.nih.gov/protein/Q0ZHF7) | 5 | 4 |
| [MavO](http://www.ncbi.nlm.nih.gov/protein/Q5ZRK2) | 5 | 0 |
| [Lem29](http://www.ncbi.nlm.nih.gov/protein/307611674), [lpg3000](http://www.ncbi.nlm.nih.gov/protein/Q5ZR83) | 4 | 4 |
| [lpg0275](http://www.ncbi.nlm.nih.gov/protein/Q5ZYT6) | 4 | 0 |
| [lpg1907](http://www.ncbi.nlm.nih.gov/protein/Q5ZUA0), [lpg2885](http://www.ncbi.nlm.nih.gov/protein/Q5ZRJ6) | 3 | 3 |
| [Ceg23](http://www.ncbi.nlm.nih.gov/protein/307610321), [LigA](http://www.ncbi.nlm.nih.gov/protein/Q9AJ94), [LtpJ](http://www.ncbi.nlm.nih.gov/protein/307611260), [SidF](http://www.ncbi.nlm.nih.gov/protein/Q5ZSD5), [lpg0963](http://www.ncbi.nlm.nih.gov/protein/Q5ZWX1), [lpw_28181](http://www.ncbi.nlm.nih.gov/protein/307611450) | 3 | 2 |
| [LegN](http://www.ncbi.nlm.nih.gov/protein/Q5ZS00), [RavP](http://www.ncbi.nlm.nih.gov/protein/Q5ZWD3), [SdbA](http://www.ncbi.nlm.nih.gov/protein/Q5ZYT6), [SidG](http://www.ncbi.nlm.nih.gov/protein/YP_095384.1), [lpg0260](http://www.ncbi.nlm.nih.gov/protein/Q5ZYV1), [lpg1137](http://www.ncbi.nlm.nih.gov/protein/Q5ZWE8), [lpp2486](http://www.ncbi.nlm.nih.gov/protein/Q5X2A7) | 3 | 0 |
| [Ceg4](http://www.ncbi.nlm.nih.gov/protein/307608842), [Ceg9](http://www.ncbi.nlm.nih.gov/protein/307609068), [Ceg15](http://www.ncbi.nlm.nih.gov/protein/Q5ZYD3), [LegC2](http://www.ncbi.nlm.nih.gov/protein/Q5ZUC3), [LegC3](http://www.ncbi.nlm.nih.gov/protein/Q5ZUU1), [LegU1](http://www.ncbi.nlm.nih.gov/protein/AAU26278.1), [PieE](http://www.ncbi.nlm.nih.gov/protein/307610687), [PpeB](http://www.ncbi.nlm.nih.gov/protein/307610398), [RavS](http://www.ncbi.nlm.nih.gov/protein/Q5ZWA2), [YlfB](http://www.ncbi.nlm.nih.gov/protein/Q5ZUC3), [lpg0518](http://www.ncbi.nlm.nih.gov/protein/Q5ZY54), [lpg1148](http://www.ncbi.nlm.nih.gov/protein/Q5ZWD7), [lpg1484](http://www.ncbi.nlm.nih.gov/protein/Q5ZVF6), [lpg1689](http://www.ncbi.nlm.nih.gov/protein/P37033), [lpg1776](http://www.ncbi.nlm.nih.gov/protein/Q5ZUL7), [lpg1959](http://www.ncbi.nlm.nih.gov/protein/Q5ZU49), [lpg2223](http://www.ncbi.nlm.nih.gov/protein/YP_096235.1), [lpg2271](http://www.ncbi.nlm.nih.gov/protein/Q5ZT91), [lpg2628](http://www.ncbi.nlm.nih.gov/protein/Q5ZS91), [lpg2692](http://www.ncbi.nlm.nih.gov/protein/Q5ZS27) | 2 | 2 |
| [LegC7](http://www.ncbi.nlm.nih.gov/protein/Q5ZT67)/[YflA](http://www.ncbi.nlm.nih.gov/protein/Q5ZT67), [LepB](http://www.ncbi.nlm.nih.gov/protein/Q5ZSM7), [Lem12](http://www.ncbi.nlm.nih.gov/protein/307610326), [RavM](http://www.ncbi.nlm.nih.gov/protein/Q5ZWH6) | 2 | 1 |
| [VpdC](http://www.ncbi.nlm.nih.gov/protein/307610129), [LegC5](http://www.ncbi.nlm.nih.gov/protein/Q5ZVF2), [lpg2505](http://www.ncbi.nlm.nih.gov/protein/Q5ZSL2) | 2 | 0 |
| [LegA7](http://www.ncbi.nlm.nih.gov/protein/Q5ZYG9), [LegK1](http://www.ncbi.nlm.nih.gov/protein/Q5ZVF7), [LegT](http://www.ncbi.nlm.nih.gov/protein/Q5ZVW1), [LegY](http://www.ncbi.nlm.nih.gov/protein/Q5ZYF0), [Lem12](http://www.ncbi.nlm.nih.gov/protein/307610326), [MavE](http://www.ncbi.nlm.nih.gov/protein/Q5ZT21), [RavR](http://www.ncbi.nlm.nih.gov/protein/Q5ZWB9), [RavT](http://www.ncbi.nlm.nih.gov/protein/Q5ZVX2), [lpg0041](http://www.ncbi.nlm.nih.gov/protein/Q5ZZG9), [lpg1578](http://www.ncbi.nlm.nih.gov/protein/Q5ZV62), [lpg1667](http://www.ncbi.nlm.nih.gov/protein/Q5ZUX5), [lpg2443](http://www.ncbi.nlm.nih.gov/protein/YP_096451.1) | 1 | 1 |
| Ceg7, Ceg18, Ceg19, CegC3, LegA6, LegA14, LegAS4, LegC6, LegL2, LegP, LegS1, Lem7, Lem19, Lem27, LpnE, MarB, RavO, RavQ, SidB, VipA, VipD, VipF, lpg0634, lpg1666, lpg1752, lpg2222 | 1 | 0 |
| SdhB, lpg0135, lpg2912 | 0 | 1 |
| Ceg3, Ceg5, Ceg7, Ceg8, Ceg10, Ceg11, Ceg14, Ceg25, Ceg28, Ceg29, Ceg30, Ceg32, Ceg33, Ceg34, CegC1, CegC2, CegC4, Leg16, Leg17, LegA1, LegA2, LegA3, LegA5, LegA8, LegA9, LegA10, LegA11, LegA12, LegA15, LegAS4, LegAU13, LegC1, LegC4, LegD2, LegG1, LegG2, LegK2, LegK3, LegL1, LegL3, LegL5, LegL7, LegLC4, LegLC8, LegS2, LegU2, Lem1, Lem2, Lem3, Lem4, Lem5, Lem6, Lem8, Lem9, Lem10, Lem11, Lem14, Lem15, Lem16, Lem17, Lem19, Lem20, Lem21, Lem22, Lem23, Lem24, Lem25, Lem26, Lem28, LepA, LidA, LirA, LirB, LirC, LirD, LirE, LirF, LtpA, LtpB, LtpC, LtpD, LtpE, LtpF, LtpG, LtpH, LtpI, MavA, MavC, MavD, MavF, MavG, MavH, MavI, MavJ, MavK, MavI, MavM, MavQ, MavV, PieF, PieG, PpgA, RalF, RavA, RavB, RavD, RavE, RavF, RavG, RavH, RavI, RavJ, RavK, RavL, RavN, RavW, RavX, RavY, RavZ, RvfA, SdbB2, SdbC, SdcA, SdeA, SdeB, SdeC, SdeD, SdhA, SdjA, SetA, SidC, SidD, SidE, SidH, SidI, SidJ, SidK, SidM/DrrA, VpdA, VpdB, WipA, WipB, and 39 ‘hypothetical proteins’. | 0 | 0 |
